# Association between genetic and socioenvironmental risk for schizophrenia during upbringing in a UK longitudinal cohort

**DOI:** 10.1017/S0033291720003347

**Published:** 2022-06

**Authors:** J. B. Newbury, L. Arseneault, A. Caspi, T. E. Moffitt, C. L. Odgers, D. W. Belsky, K. Sugden, B. Williams, A. P. Ambler, T. Matthews, H. L. Fisher

**Affiliations:** 1 King's College London, Social, Genetic & Developmental Psychiatry Centre, Institute of Psychiatry, Psychology & Neuroscience, London, UK; 2 Population Health Sciences, Bristol Medical School, University of Bristol, Bristol, UK; 3 Department of Psychology and Neuroscience, Duke University, Durham, NC, USA; 4 Department of Psychiatry and Behavioral Sciences, and Centre for Genomic and Computational Biology, Duke University, Durham, NC, USA; 5 Social Science Research Institute, Duke University, Durham, NC, USA; 6 Department of Psychological Science, School of Social Ecology, University of California, Irvine, CA, USA; 7 Department of Epidemiology and Robert N Butler Aging Center, Columbia University, Mailman School of Public Health, NY, USA; 8 ESRC Centre for Society and Mental Health, King's College London, London, UK.

**Keywords:** Childhood and adolescence, family psychiatric history, gene-environment correlation, neighborhood, polygenic risk scores, psychosis, social drift, urbanicity

## Abstract

**Background:**

Associations of socioenvironmental features like urbanicity and neighborhood deprivation with psychosis are well-established. An enduring question, however, is whether these associations are causal. Genetic confounding could occur due to downward mobility of individuals at high genetic risk for psychiatric problems into disadvantaged environments.

**Methods:**

We examined correlations of five indices of genetic risk [polygenic risk scores (PRS) for schizophrenia and depression, maternal psychotic symptoms, family psychiatric history, and zygosity-based latent genetic risk] with multiple area-, neighborhood-, and family-level risks during upbringing. Data were from the Environmental Risk (E-Risk) Longitudinal Twin Study, a nationally-representative cohort of 2232 British twins born in 1994–1995 and followed to age 18 (93% retention). Socioenvironmental risks included urbanicity, air pollution, neighborhood deprivation, neighborhood crime, neighborhood disorder, social cohesion, residential mobility, family poverty, and a cumulative environmental risk scale. At age 18, participants were privately interviewed about psychotic experiences.

**Results:**

Higher genetic risk on all indices was associated with riskier environments during upbringing. For example, participants with higher schizophrenia PRS (OR = 1.19, 95% CI = 1.06–1.33), depression PRS (OR = 1.20, 95% CI = 1.08–1.34), family history (OR = 1.25, 95% CI = 1.11–1.40), and latent genetic risk (OR = 1.21, 95% CI = 1.07–1.38) had accumulated more socioenvironmental risks for schizophrenia by age 18. However, associations between socioenvironmental risks and psychotic experiences mostly remained significant after covariate adjustment for genetic risk.

**Conclusion:**

Genetic risk is correlated with socioenvironmental risk for schizophrenia during upbringing, but the associations between socioenvironmental risk and adolescent psychotic experiences appear, at present, to exist above and beyond this gene-environment correlation.

## Background

The incidence of psychotic disorders is not evenly distributed across different geographical and social conditions, but patterned across socioenvironmental gradients such as urbanicity (van Os, Linscott, Myin-Germeys, Delespaul, & Krabbendam, 2009; Vassos, Pedersen, Murray, Collier, & Lewis, 2012), deprivation (Kirkbride, Jones, Ullrich, & Coid, 2014; O'Donoghue, Roche, & Lane, 2016), crime (Bhavsar, Boydell, Murray, & Power, 2014), and air pollution (Gao, Xu, Guo, Fan, & Zhu, 2017; Horsdal et al., 2019). Consistent with this literature, this team has previously shown that subclinical psychotic experiences, such as hearing voices and paranoia, are also more common among children and teenagers raised in urban, deprived, dangerous, and polluted environments *v.* those raised in more advantaged settings (Newbury et al., 2016, 2017, 2018, 2019; Polanczyk et al., 2010). Early psychotic experiences overlap symptomatically and etiologically with adult psychotic disorders (Polanczyk et al., 2010; Poulton et al., 2000; van Os et al., 2009), which makes them a valuable and important phenotype for researchers and clinicians.

Socioenvironmental exposures could plausibly increase the risk for psychosis via various non-mutually exclusive mechanisms, such as by promoting psychosocial stress and disrupting neurocognitive development (Alvarez, Kubzansky, Campen, & Slavich, 2018; Howes & Murray, 2014; Lederbogen et al., 2011; van Winkel, Stefanis, & Myin-Germeys, 2008). An enduring question, however, is whether associations of socioenvironmental risk factors with psychosis are truly causal. The process by which people ‘choose’ where to live is not random: it is influenced by our individual characteristics and resources. These selection processes likely result in compositional differences between individuals living in advantaged *v.* disadvantaged environments. For example, functional impairment due to high genetic risk could lead individuals to drift downward in social mobility into more crowded and impoverished settings. This selection process could occur over an individual's lifetime (intra-generational), as well as across generations (inter-generational) (Solmi, Lewis, Zammit, & Kirkbride, 2019), and thereby confound the observed associations between the environment and psychosis.

Findings from Scandinavian registry data (Mortensen et al., 1999; Pedersen, 2001) have long shown that associations between urbanicity and psychosis are not confounded by family psychiatric history, the workhorse measure of genetic risk. However, family history captures only the diagnosed fraction of genetic risk for psychopathology (Yang, Visscher, & Wray, 2010). Polygenic risk scores (PRS) – which aggregate the effects of thousands of genetic variants to index an individual's genetic risk for a phenotype (Dudbridge, 2013; Plomin, Haworth, & Davis, 2011) – offer new opportunities to examine the role of genetics in the link between socioenvironmental risk and psychosis. A handful of recent studies have explored this question. Higher schizophrenia PRS has been associated with urbanicity and deprivation in adulthood (Colodro-Conde et al., 2018; Sariaslan et al., 2016), and with deprivation and air pollution at birth (Horsdal et al., 2019; Solmi et al., 2019). In contrast, other studies have reported null associations between PRS and urbanicity at birth (Paksarian et al., 2018; Solmi et al., 2019). Furthermore, we recently identified consistent associations between PRS for education and ecological risk in childhood; but associations were inconsistent for PRS for schizophrenia (Belsky et al., 2019). Findings remain equivocal. Therefore, the present study addressed this topic by examining the association between genetic risk and socioenvironmental risk for schizophrenia during upbringing.

Like family history, PRS are imperfect measures of genetic risk. Complex traits are polygenic, and PRS currently capture only a small portion of this genetic variance (Ripke et al., 2014; Wray et al., 2018); while at the same time confirming the presence of substantial pleiotropy between different disorders like psychosis and depression (Legge et al., 2019; Pain et al., 2018). Therefore, this study incorporated five genetic risk indices, including schizophrenia PRS, depression PRS, maternal psychotic symptoms, family psychiatric history, and latent genetic risk scores derived using the twin design. This allowed us to contrast findings between PRS, which are partial through direct measures of genetic risk that are uninfluenced by the environment, with family- and twin-based measures, which capture risk more comprehensively but reflect both genetic and environmental influences. Furthermore, as well as area-level features like urbanicity, our study included detailed, high-resolution measures of neighborhood- and family-level risks that are typically enriched in cities. Based on our previous findings, measures included urbanicity, air pollution, neighborhood deprivation, neighborhood crime, neighborhood disorder, neighborhood social cohesion, residential mobility, and family poverty. Finally, rather than focusing on one time-point (e.g. birth or adulthood), our study spanned birth to age 18. Our rationale was that socioenvironmental exposures early in life, rather than in adulthood, often associate with psychosis most strongly (Pedersen & Mortensen, 2001), meaning that interventions during childhood and adolescence hold the best promise for improving mental health outcomes (Davidson, Grigorenko, Boivin, Rapa, & Stein, 2015). Additionally, children *v.* young adults have relatively little choice about where they live, meaning that intra-generational drift may increase across development. A focus from birth to adolescence will therefore highlight whether correlations between genetic and environmental risk indicate (a) reverse causation in which genetic risk leads *adults* into riskier environments, meaning that addressing risks would have little impact on psychosis outcome; or (b) that genetic risk influences exposure to causal risk factors, in which case addressing risks would reduce young people's risk for psychosis.

## Methods

### Study cohort

Participants were members of the Environmental Risk (E-Risk) Longitudinal Twin Study, which tracks the development of a nationally representative birth cohort of 2232 twin children born in 1994–1995 across England and Wales and initially assessed at age 5. Follow-up home-visits were conducted when participants were aged 7, 10, 12 and 18 (participation rates were 98%, 96%, 96% and 93%, respectively). At age 18, the E-Risk sample included 2066 participants, comprising 56% monozygotic (MZ) twin pairs and 49% males. There were no differences between those who did and did not take part at age 18 in terms of age-5 socioeconomic status (SES) (χ^2^ = 0.86; *p* = 0.65), age-5 IQ scores (*t* = 0.98; *p* = 0.33), or age-5 internalizing or externalizing behavioral problems (*t* = 0.40; *p* = 0.69 and *t* = 0.41; *p* = 0.68, respectively). E-Risk families are representative of UK households across the spectrum of neighborhood socioeconomic conditions according to consumer-classification systems (CACI Information Services, 2006): at follow-up, 27% of E-Risk participants lived in ‘wealthy achiever’ neighborhoods compared to 25% of households nationwide; 7% *v.* 12% lived in ‘urban prosperity’ neighborhoods; 28% *v.* 27% lived in ‘comfortably off’ neighborhoods; 13% *v.* 14% lived in ‘moderate means’ neighborhoods; and 26% *v.* 21% lived in ‘hard-pressed’ neighborhoods (Odgers et al., 2012*b*). Likewise, E-Risk families are representative of UK households according to the Index of Multiple Deprivation (online Supplementary Figure 1). The Joint South London and Maudsley and the Institute of Psychiatry Research Ethics Committee approved each phase of the study. Parents gave written informed consent and participants gave written assent at ages 5–12 and written informed consent at age 18. Further details about the sample are reported elsewhere (Moffitt, 2002) and in the Supplementary Materials. Table 1 displays characteristics for the analysis sample, together with information on missing data.

**Table 1.** Sample characteristics and missing data

Characteristics and covariates	*M*/*N*	s.d. / %	Range	Missing *N* (%)
Sex				
Male	986	49.32	–	0
Female	1013	50.68	–	
Zygosity				
Monozygotic	1110	55.53	–	0
Dizygotic	889	44.47	–	
Schizophrenia PRS	0	1	−3.75–3.53	0
Depression PRS	0	1	−3.31–3.39	0
Maternal psychotic symptoms	0.30	0.80	0–5	79 (3.95)
Family psychiatric history	0.37	0.27	0–1	79 (3.95)
Latent genetic risk				
0	743	39.97	–	140 (7.00)
1	566	30.45	–	
2	248	13.34	–	
3	302	16.25	–	
Urbanicity (age 5)				
Rural	395	20.86	–	104 (5.25)
Intermediate	932	49.21	–	
Urban	567	29.94	–	
Urbanicity (age 12)				
Rural	416	21.64	–	77 (3.85)
Intermediate	966	50.26	–	
Urban	540	28.10	–	
Urbanicity (age 18)				
Rural	354	21.07	–	319 (15.96)
Intermediate	849	50.54	–	
Urban	477	28.39	–	
Air pollution (age 10)	25.14	9.78	2.59–57.87	71 (3.52)
Air pollution (age 18)	18.65	8.81	2.03–69.48	179 (8.95)
Neighborhood deprivation (age 5)				
Wealthy achiever	454	23.20	–	42 (2.10)
Urban prosperity	90	4.60	–	
Comfortably off	568	29.02	–	
Moderate means	289	14.77	–	
Hard pressed	556	28.41	–	
Neighborhood deprivation (age 12)				
Wealthy achiever	506	26.31	–	76 (3.80)
Urban prosperity	92	4.78	–	
Comfortably off	581	30.21	–	
Moderate means	243	12.64	–	
Hard pressed	501	26.05	–	
Neighborhood deprivation (age 18)				
Wealthy achiever	445	27.07	–	355 (17.76)
Urban prosperity	110	6.69	–	
Comfortably off	460	2.98	–	
Moderate means	207	12.59	–	
Hard pressed	422	25.67	–	
Neighborhood crime	214.42	227.73	1.00–1780.75	82 (4.10)
Neighborhood disorder	0.47	0.34	0–1.93	64 (3.20)
Neighborhood social cohesion	2.24	0.50	0–3.71	64 (3.20)
Residential mobility (5-12)				
0 moves	1062	56.31	–	113 (5.65)
1 move	479	25.40	–	
2 + moves	345	18.29	–	
Residential mobility (5-18)				
0 moves	803	44.91	–	211 (10.56)
1 move	500	27.96	–	
2 + moves	485	27.13	–	
Family poverty				
High SES	666	33.32	–	0
Medium SES	662	33.12	–	
Low SES	671	33.57	–	

Note: M, mean; N, number; PRS, polygenic risk score; s.d., standard deviation; SES, socioeconomic status.

### Measures

#### Genotyping participants

We used Illumina HumanOmni Express 24 BeadChip arrays (Version 1.1; Illumina, Hayward, CA) to assay common single-nucleotide polymorphism (SNP) variation in the genomes of cohort members. The resulting database was restricted to SNPs called successfully in >98% of the cohort and in Hardy-Weinberg equilibrium (*p* > 0.001). We imputed additional SNPs using the IMPUTE2 software (Version 2.3.1; https://mathgen.stats.ox.ac.uk/impute/impute_v2.html; Howie, Donnelly, & Marchini, 2009) and the 1000 Genomes Phase 3 reference panel (Genomes Project Consortium, 2015). Imputation was conducted on autosomal SNPs appearing in dbSNP (Version 140; http://www.ncbi.nlm.nih.gov/SNP/; Sherry et al., 2001) that were ‘called’ in more than 98% of the samples. Invariant SNPs were excluded. The E-Risk cohort contains MZ twins, who are genetically identical; we therefore empirically measured genotypes of one randomly selected twin per pair and assigned these data to their MZ co-twin. We directly measured genotypes of both members of dizygotic (DZ) twin pairs. Prephasing and imputation were conducted using a 50-million-base-pair sliding window. The resulting genotype databases included genotyped SNPs and SNPs imputed with 90% probability of a specific genotype among the European descent members of the E-Risk cohort (*N* = 1999 participants in 1011 families). Note that the analysis sample for the present study included only ethnically White participants because PRS currently has uncertain validity in non-European descent populations (Martin et al., 2017; Vassos et al., 2017).

#### Polygenic risk scores

PRS were created for European descent E-Risk participants following the method described by Dudbridge (2013) using PRSice (Version 1.22; http://prsice.info/; Euesden, Lewis, and O'Reilly, 2014). For schizophrenia PRS, SNPs reported in the results of the latest GWAS for schizophrenia (Pardiñas et al., 2018) were matched with SNPs in the E-Risk cohort, irrespective of nominal significance with schizophrenia. We then performed clumping by retaining the SNP with the smallest *p* value from each LD block (excluding SNPs with *r*^2^ > 0.1 in 500-kb windows), then weighted retained SNPs by effect estimate. To control for possible population stratification, we conducted a principal component analysis of our genome-wide SNP database using PLINK (Version 1.9; Chang et al., 2015). One twin was selected at random from each family for principal component analysis. SNP loadings for principal components were applied to co-twin genetic data to compute principal component values for the full sample. The 10 principal components explained 2.8% of the variance in the schizophrenia PRS. We residualized polygenic scores for the first 10 principal components estimated from the genome-wide SNP data. The residualized scores were normally distributed. We standardized residuals for analysis (*M* = 0, s.d. = 1, range = −3.75–3.53). For depression PRS, SNPs reported in the latest GWAS for major depression (Wray et al., 2018) were matched with SNPs in the E-Risk cohort, irrespective of nominal significance with depression. Clumping and principal components analysis were conducted as above. Residualized scores were normally distributed, and these were once again standardized for analysis (*M* = 0, s.d. = 1, range = −3.31–3.39).

#### Maternal psychotic symptoms and family psychiatric history

Maternal psychotic symptoms and family history (Table 1) were assessed in private interviews with the mothers when children were aged 12. For maternal psychotic symptoms, mothers were interviewed using the Diagnostic Interview Schedule (DIS; Robins, Cottler, Bucholz, and Compton, 1995) for DSM-IV (American Psychiatric Association, 1994) which provides a symptom count for characteristic symptoms of schizophrenia (e.g. hallucinations, delusions, anhedonia). For family psychiatric history, mothers reported on their own mental health history and the mental health history of their biological mothers, fathers, sisters, brothers, and the twins' biological father (Weissman et al., 2000). This was converted into the proportion of family members with a history of psychiatric disorder (Milne et al., 2008).

#### Latent genetic risk scores

Latent genetic risk scores were derived based on each participant's zygosity and whether their co-twin had psychotic experiences. As described by Arseneault et al. (2011), each twin is represented in the data twice, first as the target and then as the co-twin. A continuum of genetic risk is generated, on the basis that MZ twins are ~100% genetically identical, whereas DZ twins share ~50% of the same genes. Thus, we assigned a score of 0 to participants if their MZ co-twin *did not* have psychotic experiences; a score of 1 to participants if their DZ co-twin *did not* have psychotic experiences; a score of 2 to participants if their DZ twin *did* have psychotic experiences; and a score of 3 to participants if their MZ twin *did* have psychotic experiences.

#### Adolescent psychotic experiences

At age 18, each E-Risk participant was privately interviewed by a research worker about 13 psychotic experiences occurring since age 12. Seven items referred to delusions and hallucinations (Polanczyk et al., 2010), such as ‘have you ever thought you were being watched, followed or spied on?’ and ‘do you hear voices that others cannot?’. Six items referred to unusual experiences which drew on item pools since formalized in prodromal psychosis instruments including the PRIME-screen and SIPS (Loewy, Pearson, Vinogradov, Bearden, & Cannon, 2011), such as ‘I believe I have special abilities or powers beyond my natural talents’. Further information on this measure is provided in Supplementary Materials. Research workers coded each item 0, 1, or 2 indicating respectively: ‘not present’, ‘probably present’ and ‘definitely present’. All 13 items were summed (*M* = 1.19, s.d. = 2.58, range = 0–18), and scores were placed into an ordinal scale, corresponding to the number of different psychotic experiences rather than frequency of occurrence. Just over 30% of participants had at least one psychotic experience between ages 12 and 18: 69.8% reported no psychotic experiences (coded 0; *n* = 1440), 15.5% reported one or two psychotic experiences (coded 1; *n* = 319), 8.1% reported three–five psychotic experiences (coded 2; *n* = 166), and 6.7% reported six or more psychotic experiences (coded 3; *n* = 138). This is similar to the prevalence of psychotic experiences self-reported in other general population non-twin samples (Spauwen, Krabbendam, Lieb, Wittchen, & van Os, 2004; Yoshizumi, Murase, Honjo, Kaneko, & Murakami, 2004; Yung et al., 2009).

### Socioenvironmental risks

**Fig. 1.**
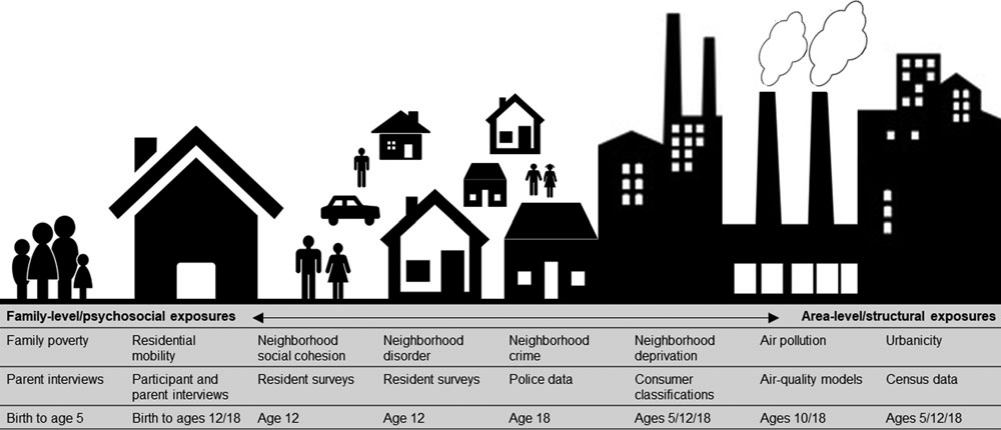
Illustration of the nature and source of environmental risk variables used in this study.

Socioenvironmental risk variables are described below, in Fig. 1, and in the Supplementary Materials. Some variables were on ordinal scales and some were continuous. All continuous variables were placed into ordinal scales to allow consistent analyses and comparison between variables. Categorizations were based on official classifications and/or precedent. Urbanicity, neighborhood deprivation, and air pollution were available at several time-points. Therefore, we included data from multiple time-points across childhood and adolescence to explore temporal changes in associations.

*Urbanicity* was derived from classifications from 2011 census data (Office for National Statistics, 2013) and linked to participants' home postcodes at ages 5, 12, and 18. Classifications incorporate data on residential density, settlement size and population sparsity. Resolution is at the Lower Layer Super Output Area (LSOA) level, which contain an average of 1500 residents. A three-level urbanicity variable was used representing rural, intermediate and urban settings (Table 1). E-Risk participants are nationally-representative in terms of ONS urbanicity classifications; for instance, the nationwide distribution of rural, intermediate, and urban residents in the UK is 18.9%, 45.0%, and 36.1%, respectively.

*Air pollution* exposure was measured using a coupled regional chemical transport model and street-scale dispersion model (Beevers, Kitwiroon, Williams, & Carslaw, 2012; Carslaw, 2011). Based on our previous findings in this cohort (Newbury et al., 2019), the present study focusses on nitrogen dioxide (NO_2_) exposure. Annualized estimates of ambient NO_2_ were linked to participants' home postcodes at ages 10 and 18. The model achieves 20 × 20 meter resolution. For this study, annualized NO_2_ exposure was categorized into quartiles (1 = least polluted; 4 = most polluted).

*Neighborhood deprivation* was constructed using A Classification of Residential Neighborhoods (ACORN), a geodemographic discriminator developed by CACI Information Services (CACI Information Services; http://www.caci.co.uk/). Detailed information about ACORN's classification of neighborhood-level socioeconomic-status (SES) has been provided previously (Caspi, Taylor, Moffitt, & Plomin, 2000; Odgers et al., 2009, 2012*b*). Briefly, CACI utilized over 400 census variables for Great Britain (e.g. educational qualifications, unemployment, housing tenure) and CACI's consumer lifestyle database. Classifications ranged from ‘Wealthy Achiever’ (coded 1) to ‘Hard Pressed’ (coded 5) neighborhoods (Table 1). Each family in our sample was matched to the ACORN code for its neighborhood via postcodes at ages 5, 12, and 18 (Caspi et al., 2000).

*Neighborhood crime* was measured using police data for England and Wales for 2011 and linked to participants' home postcodes at age 18. The total number of crimes occurring each month within a one-mile radius of each participant's home was tallied (Table 1). Scores were placed into quartiles for analysis (1 = lowest crime; 4 = highest crime).

*Neighborhood disorder and social cohesion* were measured via resident reports sent to >5000 immediate neighbors of participants in 2008 (Odgers et al., 2009; Odgers, Caspi, Bates, Sampson, & Moffitt, 2012*a*). Items enquired about physical and social signs of threat in the neighborhood (neighborhood disorder) and the quality and quantity of social interactions between neighbors (social cohesion). Items were averaged across each neighborhood characteristic and across respondents from the same neighborhood, and linked to home postcodes at age 12 (Table 1). Scores were grouped into tertiles for analysis at the 33^rd^ and 66^th^ centile (1 = lowest neighborhood disorder/highest social cohesion; 3 = highest neighborhood disorder/lowest social cohesion).

*Residential mobility* was measured from birth to age 18 via parent (ages 5–12) and participant (age 18) interviews. Residential moves were summed across phases 5–12 and phases 5–18, and collapsed into three categories including 1 (no moves), 2 (1 move), and 3 (2 or more moves) (Table 1).

*Family poverty* (SES) was measured as a composite of parental income, education, and occupation via interviews with mothers when children were aged 5. The latent variable was categorized into tertiles (i.e. low-, medium-, and high-SES; Trzesniewski, Moffitt, Caspi, Taylor, and Maughan, 2006; Table 1). The present study uses a reverse-scored measure from 1 (high-SES) to 3 (low-SES).

#### Cumulative environmental risk scale

To compare participants with the most *v.* least disadvantaged upbringings we created a cumulative environmental risk scale by summing the top scores of each of the socioenvironmental variables. For analyses with the genetic risk indices, the variable included data up to age 18. For analyses with psychotic experiences, the variable included data only up to age 12 to ensure temporal ordering of exposures and outcome. For risk factors measured at multiple phases (urbanicity, deprivation, NO_2_) we averaged scores across phases prior to constructing the cumulative scale. Percentages of participants exposed to 0, 1, 2, 3, 4 or 5 or more risks up to age 12 were: 26.5%, 20.8%, 16.7%, 15.2%, 10.9% and 10.0%; and up to age 18 were; 22.5%, 21.0%, 15.8%, 13.7%, 11.4%, and 15.6%, respectively (scores of 5–8 risks were collapsed due to small numbers within these categories).

### Statistical analyses

We conducted the analyses using Stata (v16.0) following three steps. First, we used ordinal logistic regression to examine associations of the genetic risk indices with adolescent psychotic experiences. Second, we used ordinal logistic regression to examine associations of the genetic risk indices with the socioenvironmental risk variables, with the genetic risk indices treated as the independent variables. For step 2, one twin per each MZ twin pair was randomly dropped, because MZ twins correlate ~100% for their genetic sequence and typically share the same neighborhood environments during childhood. Alongside steps 1 and 2, we plotted genetic risk Z-scores across psychotic experience scores and the cumulative environmental risk scale. Third, we used ordinal logistic regression to examine the associations of socioenvironmental risks from birth to age 12 with adolescent psychotic experiences and added the genetic risk indices as covariates to examine attenuation of effects. All genetic risk indices were standardized for analysis (i.e. *M* = 0, s.d. = 1) to allow comparison between measures. Steps 1–3 accounted for the non-independence of twin observations using the cluster command in Stata. This procedure is derived from the Huber-White variance estimator and provides robust standard errors adjusted for within-cluster correlated data (Rogers, 1994). Analyses were conducted following multiple imputation using chained equations to handle missing covariate data (described further in the Supplementary Materials). Multiple imputations were only conducted for the 1999 White, genotyped participants because schizophrenia PRS currently has uncertain validity in non-European descent populations (Martin et al., 2017; Vassos et al., 2017). Complete case analyses are reported in the Supplementary Materials.

## Results

### Is genetic risk associated with adolescent psychotic experiences?

**Fig. 2.**
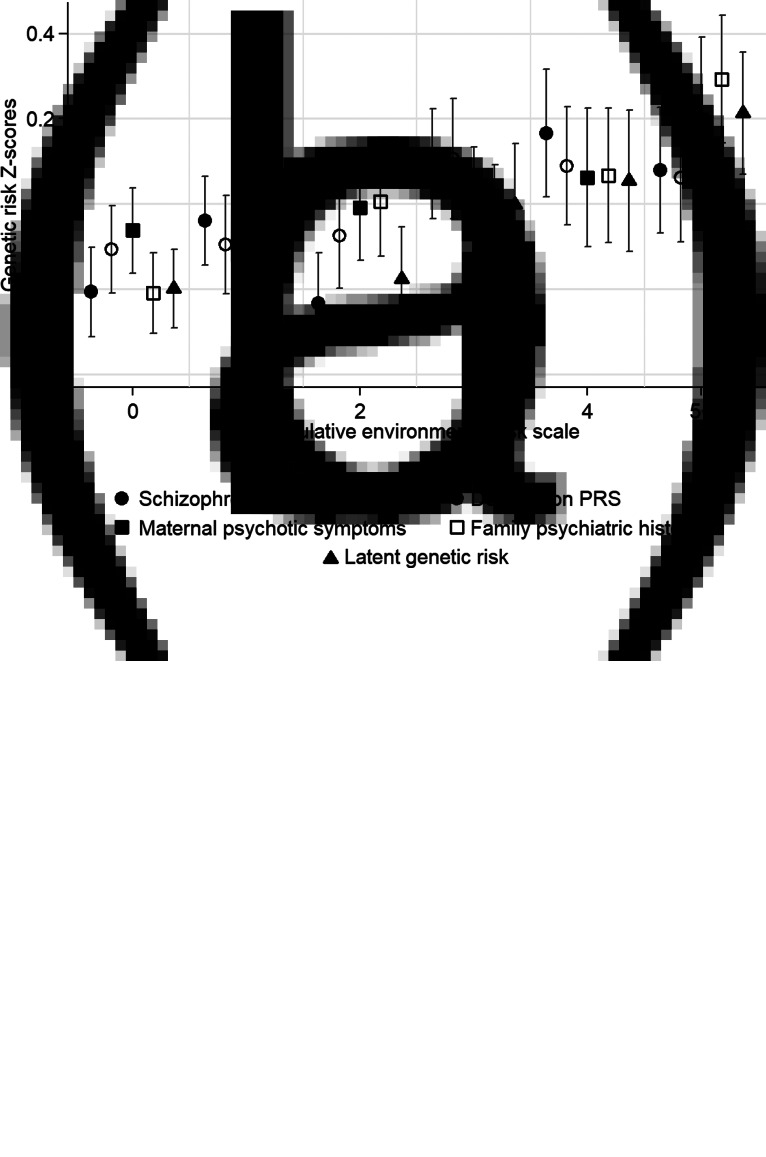
Genetic risk Z-scores and 95% confidence intervals (indicated by double-headed lines) across (a) adolescent psychotic experiences scores, and (b) the cumulative environmental risk scale. Note: PRS, polygenic risk score.

Adolescents with higher depression PRS (OR = 1.17, 95% CI = 1.05–1.31, *p* = 0.005), maternal psychotic symptoms (OR = 1.15, 95% CI = 1.05–1.26, *p* = 0.002), family psychiatric history (OR = 1.32, 95% CI = 1.18–1.47, *p* < 0.001) and latent genetic risk (OR = 1.71, 95% CI = 1.49–1.96, *p* < 0.001) tended to report more psychotic experiences than those with lower genetic risk. However, the association between schizophrenia PRS and adolescent psychotic experiences failed to meet conventional levels of statistical significance (OR = 1.12, 95% CI = 1.00–1.25, *p* = 0.052). Figure 2*a* illustrates these associations with genetic risk Z-scores plotted across psychotic experience scores.

### Is genetic risk associated with socioenvironmental risk for schizophrenia during upbringing?

Figure 2*b* plots genetic risk *Z*-scores across the cumulative environmental risk scale and shows that participants with higher genetic risk tended to experience more socioenvironmental risks for schizophrenia during upbringing. Table 2 displays odds ratios for these associations. Schizophrenia PRS was associated at *p* < 0.05 with the cumulative environmental risk scale, age-18 urbanicity, neighborhood crime, neighborhood disorder, residential mobility, and family poverty (all OR's >1.12). Depression PRS was associated at *p* < 0.05 with the cumulative environmental risk scale, age-12 and age-18 neighborhood deprivation, neighborhood crime, and neighborhood social cohesion (all ORs >1.11). Maternal psychotic symptoms were associated at *p* < 0.05 only with residential mobility and family poverty (all OR's >1.18). Family psychiatric history was associated at *p* < 0.05 with the cumulative environmental risk scale, age-12 and age-18 neighborhood deprivation, neighborhood disorder, residential mobility, and family poverty (all OR's >1.15). Finally, latent genetic risk was associated at *p* < 0.05 with the cumulative environmental risk scale, age-5 and age-18 neighborhood deprivation, neighborhood crime, residential mobility, and family poverty (all ORs >1.12).

**Table 2.** Association of genetic risk indices with socioenvironmental risk factors for schizophrenia

Socioenvironmental risk factors	Genetic risk indices				
	Schizophrenia PRS	Depression PRS	Maternal psychotic symptoms	Family psychiatric history	Latent genetic risk
	OR (95% CI)	OR (95% CI)	OR (95% CI)	OR (95% CI)	OR (95% CI)
Environmental risk scale (age 5–18)	1.18** (1.06–1.33)	1.20** (1.08–1.34)	1.08 **(**0.96–1.23**)**	1.25*** (1.11–1.40)	1.21** (1.07–1.38)
Urbanicity					
Age 5	1.10† **(**0.99–1.23**)**	1.05 **(**0.93–1.18**)**	0.90† **(**0.82–1.00**)**	1.07 **(**0.95–1.21**)**	1.10 **(**0.98–1.24**)**
Age 12	1.11† **(**0.99–1.24**)**	1.02 (0.90–1.15)	0.93 **(**0.83–1.04**)**	1.03 **(**0.91–1.15**)**	1.12† **(**1.00–1.26**)**
Age 18	1.14* (1.02–1.27)	1.07 **(**0.95–1.20**)**	0.91† **(**0.81–1.01**)**	1.03 **(**0.91–1.17**)**	1.09 **(**0.97–1.22**)**
Air pollution					
Age 10	1.06 **(**0.95–1.18**)**	1.09 **(**0.98–1.22**)**	1.00 **(**0.90–1.10**)**	1.07 **(**0.95–1.21**)**	1.09 **(**0.98–1.23**)**
Age 18	1.07 **(**0.96–1.20**)**	1.11† **(**1.00–1.24**)**	0.96 **(**0.86–1.07**)**	1.01 **(**0.89–1.14**)**	1.11† **(**0.99–1.24**)**
Neighborhood deprivation					
Age 5	1.07 **(**0.97–1.19**)**	1.08 **(**0.97–1.21**)**	1.05 **(**0.93–1.19**)**	1.07 **(**0.96–1.20**)**	1.13* (1.00–1.27)
Age 12	1.07 **(**0.97–1.19**)**	1.11* (1.00–1.24)	1.11† **(**0.99–1.25**)**	1.15* (1.03–1.29)	1.12† **(**1.00–1.25**)**
Age 18	1.01 **(**0.91–1.12**)**	1.17** (1.05–1.30)	1.07 **(**0.97–1.18**)**	1.15* (1.03–1.29)	1.15* (1.02–1.29)
Neighborhood crime (age 18)	1.13* (1.01–1.28)	1.12* (1.00–1.24)	1.08 **(**0.96–1.22**)**	1.10 **(**0.98–1.25**)**	1.12* (1.00–1.26)
Neighborhood disorder (age 12)	1.16** (1.04–1.30)	1.11† **(**1.00–1.24**)**	1.02 **(**0.89–1.16**)**	1.15* (1.02–1.30)	1.13† **(**0.99–1.28**)**
Neighborhood social cohesion (age 12)	1.10 **(**0.98–1.23**)**	1.14* (1.02–1.28)	0.97 **(**0.86–1.09**)**	1.11† **(**0.98–1.25**)**	1.10 **(**0.97–1.25**)**
Residential mobility					
Ages 5–12	1.16* (1.02–1.32)	1.06 **(**0.93–1.21**)**	1.19** (1.05–1.35)	1.40*** (1.22–1.59)	1.13† **(**0.99–1.28**)**
Ages 5–18	1.18** (1.05–1.32)	1.08 **(**0.96–1.22**)**	1.27*** (1.14–1.41)	1.38*** (1.22–1.56)	1.16* (1.02–1.33)
Family poverty (age 5)	1.12* (1.00–1.24)	1.11† **(**1.00–1.24**)**	1.18** (1.05–1.33)	1.30*** (1.14–1.47)	1.14* (1.02–1.30)

Note: CI, confidence intervals; OR, odds ratio; PRS, polygenic risk score; † *p* < 0.1; * *p* < 0.05; ** *p* < 0.01; *** *p* < 0.001. Bold font indicates *p* < 0.05. All analyses control for the non-independence of twin observations and include only one twin per MZ twin pair. Analyses were conducted following multiple imputations via chained equations for genotyped, European ancestry participants (*N* = 1999).

### Are associations between socioenvironmental risks and adolescent psychotic experiences attenuated by covariate adjustment for genetic risk?

Associations between socioenvironmental risks and adolescent psychotic experiences are reported in Table 3 and online Supplementary Table 1. Children exposed to the highest levels of each risk factor had greater odds for psychotic experiences compared to children with the lowest exposure to these risks (all unadjusted ORs = 1.43–2.98; all *p*s < 0.05). Effect sizes were slightly reduced following covariate adjustment for schizophrenia PRS (2–9% reduction), depression PRS (2–12% reduction) and maternal psychotic symptoms (0–12% reduction), and were often reduced more substantially following covariate adjustment for family history (0–45% reduction) and latent genetic risk (8–29% reduction). Effect sizes were reduced by 15% (urbanicity) to 75% (residential mobility) after simultaneous adjustment for all genetic risk indices together. Nevertheless, associations mostly remained significant.

**Table 3.** Association of socioenvironmental risk factors with adolescent psychotic experiences

Socioenvironmental risk factors^a^	Model adjustment						
	Unadjusted	Schizophrenia PRS	Depression PRS	Maternal psychotic symptoms	Family psychiatric history	Latent genetic risk	All genetic risk indices together
	OR (95% CI)	OR (95% CI)	OR (95% CI)	OR (95% CI)	OR (95% CI)	OR (95% CI)	OR (95% CI)
Environmental risk scale	2.98*** (1.88–4.73)	2.93*** (1.85–4.64)	2.88*** (2.81–4.58)	2.91*** (1.84–4.62)	2.70*** (1.71–4.25)	2.64*** (1.76–3.98)	2.34*** (1.55–3.54)
Urbanicity (age 5)	1.49* (1.06–2.08)	1.46* (1.04–2.04)	1.47* (1.05–2.05)	1.50* (1.07–2.11)	1.46* (1.04–2.04)	1.43* (1.06–1.92)	1.39* (1.03–1.87)
Urbanicity (age 12)	1.51* (1.09–2.09)	1.48* (1.07–2.05)	1.50* (1.08–2.08)	1.52* (1.10–2.11)	1.51* (1.10–2.09)	1.43* (1.08–1.90)	1.42* (1.06–1.90)
Air pollution	1.43* (1.06–1.93)	1.41* (1.04–1.91)	1.40* (1.04–1.89)	1.40* (1.04–1.90)	1.40* (1.04–1.90)	1.33* (1.02–1.72)	1.27† (0.98–1.66)
Neighborhood deprivation (age 5)	1.87*** (1.38–2.55)	1.85*** (1.36–2.52)	1.85*** (1.36–2.53)	1.85*** (1.36–2.52)	1.81*** (1.33–2.47)	1.75*** (1.34–2.30)	1.69*** (1.29–2.23)
Neighborhood deprivation (age 12)	2.14*** (1.56–2.93)	2.11*** (1.54–2.90)	2.09*** (1.53–2.87)	2.09*** (1.53–2.87)	2.04*** (1.49–2.78)	2.02*** (1.54–2.66)	1.89*** (1.43–2.50)
Neighborhood disorder	1.64** (1.24–2.18)	1.61** (1.21–2.14)	1.61** (1.21–2.13)	1.62** (1.23–2.15)	1.57** (1.19–2.08)	1.56** (1.21–2.01)	1.47** (1.14–1.90)
Neighborhood social cohesion	1.55** (1.19–2.02)	1.53** (1.18–1.99)	1.50** (1.15–1.96)	1.55** (1.19–2.01)	1.51** (1.16–1.97)	1.49*** (1.19–1.86)	1.42** (1.13–1.79)
Residential mobility	1.31* (1.00–1.71)	1.28† (0.98–1.68)	1.27† (0.97–1.67)	1.27† (0.97–1.67)	1.16 (0.88–1.54)	1.21† (0.97–1.53)	1.07 (0.85–1.36)
Family poverty	2.17*** (1.65–2.85)	2.14*** (1.63–2.82)	2.13*** (1.62–2.80)	2.10*** (1.60–2.77)	2.01*** (1.52–2.65)	2.02*** (1.60–2.55)	1.85*** (1.45–2.35)

Note: CI, confidence intervals; OR, odds ratio from ordinal logistic regression; †*p* < 0.1; *p < 0.05; **p < 0.01; ***p < 0.001.

a Odds ratios are for the top *v.* bottom level of each socioenvironmental risk factor (association across all levels are shown in online Supplementary Table 1). Aside from the unadjusted model, all models control for sex in addition to the specified genetic risk measure. Missing values were multiply imputed using chained equations for genotyped European ancestry participants (*N* = 1999). All analyses control for the non-independence of twin observations.

### Complete case analyses

Complete case analyses revealed similar point estimates (online Supplementary Tables 2 and 3).

## Discussion

In a cohort of British youth followed from birth to age 18, participants at higher genetic risk were exposed to more socioenvironmental risk factors for schizophrenia during upbringing. Associations in terms of elevated odds were apparent for almost all risk factors examined. Beyond urbanicity, our study incorporated detailed neighborhood- and family-level risks measured from multiple sources. Our sample also captures the full range of socioeconomic and neighborhood conditions found in the UK, and we were thus able to contrast high- and low-risk groups. In doing so, we found that youth with higher genetic risk tended to grow up in more deprived neighborhoods with higher crime and disorder, and within poorer families who moved house more frequently. In fact, gene-environment correlations were more consistent for neighborhood- and family-level risks compared to the area-level risks. This could suggest that gene-environment correlations at the area-level comprise more pronounced gene-environment correlations at the neighborhood- and family-level. It could also be that larger sample sizes than ours are required to powerfully detect gene-environment correlations at the area-level. Therefore, future research into geosocial patterning of genetic risk requires both precise measures of the environment and representative samples that include high-risk groups.

There was tentative evidence that gene-environment correlations increased across development. For instance, schizophrenia PRS was associated with urbanicity at age 18, but not at ages 5 or 12; and depression PRS and family history were associated with deprivation at ages 12 and 18, but not at age 5. Our findings are consistent with those from a previous report in which schizophrenia PRS was associated with urbanicity at age 15 but not urbanicity at birth (Paksarian et al., 2018). These findings provide support for a process of intra-generational drift, in which gene-environment correlations increase over time due to selection into different geographical and social conditions. A process of intra-generational drift is also supported by our novel finding that genetic risk is associated with more residential mobility during upbringing. Continued follow-up of the E-Risk cohort will allow us to test for downward drift further into adulthood. However, effect size differences across time-points were small and should be interpreted with caution. Furthermore, given that several risk factors were measured early in development, our findings cannot entirely be explained by intra-generational drift. Instead, our findings also support an inter-generational process in which families at higher genetic risk drift downward in social mobility across generations into more disadvantaged environments. That is, participants who inherited higher genetic risk also tended to be born and raised in riskier environments.

Following covariate adjustment for the genetic risk indices, effect sizes were often attenuated more substantially by family history and latent genetic risk *v.* PRS. This likely reflects, in part, that family- and twin-based measures capture genetic risk more comprehensively at present compared to PRS. This could also be because family history captures unmeasured environmental risks shared between family members which contributed to the expression of mental illness in the family. Nevertheless, the association between socioenvironmental risk factors and psychotic experiences existed above and beyond five genetic risk indices in our study. Though residual confounding is inevitable, our findings lend support for an important role of socioenvironmental conditions during upbringing in early expressions of psychosis.

### Limitations

We acknowledge several limitations. First, PRS for schizophrenia currently capture ~30% of the SNP-based heritability for schizophrenia (Ripke et al., 2014), despite behavioral genetics studies demonstrating that >70% of schizophrenia variance is due to genetic factors (Lichtenstein et al., 2009). Indeed, schizophrenia PRS was only weakly associated with adolescent psychotic experiences in our study. The ability of PRS to capture genetic liability and predict phenotypic variance is expected to increase as the size of GWAS samples increases (Visscher et al., 2017). Second, family psychiatric history (and by design, maternal psychotic symptoms) was biased towards maternal family history (though mothers also reported on the twins' father's psychiatric history). In large cohort studies with finite resources it is common to focus on maternal reports, partly because mothers typically retain main custody in instances of parental separation (Braver, Ellman, Votruba, & Fabricius, 2011). Third, it was necessary to restrict all analyses (MICE and complete case) to European descent participants because PRS are currently suitable only within ancestries matched to the original GWAS (Martin et al., 2017; Vassos et al., 2017). Fourth, adolescent psychotic experiences were self-reported and may have included false positives. In addition, the measure covered only positive psychotic symptoms. Negative and disorganized symptoms are potentially more clinically-pertinent (Ho, Nopoulos, Flaum, Arndt, & Andreasen, 1998), but are also, unfortunately, harder to assess (Stahl & Buckley, 2007). Finally, the E-Risk sample comprises twins, who could differ from singletons in terms of both measured and unmeasured factors. However, E-Risk families are representative of the UK population in terms of urbanicity (Office for National Statistics, 2013) and socioeconomic conditions (CACI Information Services, 2006; Department for Communities and Local Government, 2015). The prevalence of psychotic experiences in our sample was also comparable to those from general population studies of non-twins (Spauwen et al., 2004; Yoshizumi et al., 2004; Yung et al., 2009).

## Conclusion

Urbanicity, deprivation, neighborhood crime and disorder, residential mobility and poverty have been consistently associated with both clinical and subclinical expressions of psychosis. Our findings have implications for interpreting the causality of these associations. It is very unlikely that associations are entirely confounded by genetics. Indeed, associations between socioenvironmental risk factors and adolescent psychotic experiences existed above and beyond five indices of genetic risk in our study. However, our findings add to growing evidence that a degree of genetic confounding exists. Future investigations into the environment and psychosis should continue to consider gene-environment correlation as a non-causal contributor to associations. Understanding the processes leading to intra- and inter-generational drift could also open up valuable new avenues for improving psychiatric interventions and reducing socioenvironmental inequalities.

## Supplementary material

For supplementary material accompanying this paper visit https://doi.org/10.1017/S0033291720003347. click here to view supplementary material

## References

[ref1] Alvarez, H. A. O., Kubzansky, L. D., Campen, M. J., & Slavich, G. M. (2018). Early life stress, air pollution, inflammation, and disease: An integrative review and immunologic model of social-environmental adversity and lifespan health. Neuroscience and Biobehavioral Reviews, 92, 226–242. doi:10.1016/j.neubiorev.2018.06.002. 29874545 PMC6082389

[ref2] American Psychiatric Association (1994). Diagnostic and statistical manual of mental disorders. Washington, DC: American Psychiatric Association.

[ref3] Arseneault, L., Cannon, M., Fisher, H. L., Polanczyk, G., Moffitt, T. E., & Caspi, A. (2011). Childhood trauma and children's emerging psychotic symptoms: A genetically sensitive longitudinal cohort study. American Journal of Psychiatry, 168, 65–72. doi:10.1176/appi.ajp.2010.10040567. 20952460 PMC3536053

[ref4] Beevers, S. D., Kitwiroon, N., Williams, M. L., & Carslaw, D. C. (2012). One way coupling of CMAQ and a road source dispersion model for fine scale air pollution predictions. Atmospheric Environment, 59, 47–58. doi:10.1016/j.atmosenv.2012.05.034. 23471172 PMC3587455

[ref5] Belsky, D. W., Caspi, A., Arseneault, L., Corcoran, D. L., Domingue, B. W., Harris, K. M., … Prinz, J. (2019). Genetics and the geography of health, behaviour and attainment. Nature Human Behaviour, 3, 576–586. doi:10.1038/s41562-019-0562-1. PMC6565482 30962612

[ref6] Bhavsar, V., Boydell, J., Murray, R., & Power, P. (2014). Identifying aspects of neighbourhood deprivation associated with increased incidence of schizophrenia. Schizophrenia Research, 156, 115–121. doi:10.1016/j.schres.2014.03.014. 24731617

[ref7] Braver, S. L., Ellman, I. M., Votruba, A. M., & Fabricius, W. V. (2011). Lay judgments about child custody after divorce. Psychology, Public Policy, and Law, 17, 212. doi:10.1037/a0023194.

[ref8] CACI Information Services (2006). ACORN User guide. London, UK: CACI.

[ref9] Carslaw D. C. (2011, April 15). Defra urban model evaluation analysis - Phase 1. Report to Defra, Environmental Research Group. Retrieved from https://uk-air.defra.gov.uk/library/reports?report_id=654.

[ref10] Caspi, A., Taylor, A., Moffitt, T. E., & Plomin, R. (2000). Neighborhood deprivation affects children's mental health: Environmental risks identified in a genetic design. Psychological Science, 11, 338–342. doi:10.1111/1467-9280.00267. 11273396

[ref11] Chang, C. C., Chow, C. C., Tellier, L. C., Vattikuti, S., Purcell, S. M., & Lee, J. J. (2015). Second-generation PLINK: Rising to the challenge of larger and richer datasets. Gigascience, 4, s13742–s13015. doi:10.1186/s13742-015-0047-8. PMC4342193 25722852

[ref12] Colodro-Conde, L., Couvy-Duchesne, B., Whitfield, J. B., Streit, F., Gordon, S., Kemper, K. E., … de Zeeuw, E. L. (2018). Association between population density and genetic risk for schizophrenia. JAMA Psychiatry, 75, 901–910. doi:10.1001/jamapsychiatry.2018.1581. 29936532 PMC6142911

[ref13] Davidson, L. L., Grigorenko, E. L., Boivin, M. J., Rapa, E., & Stein, A. (2015). A focus on adolescence to reduce neurological, mental health and substance-use disability. Nature, 527, S161–S166. doi:10.1038/nature16030. 26580322

[ref14] Department for Communities and Local Government (2015). The English indices of deprivation: 2015. London: Department for Communities and Local Government.

[ref15] Dudbridge, F. (2013). Power and predictive accuracy of polygenic risk scores. Plos Genetics, 9, e1003348. doi:10.1371/journal.pgen.1003348. 23555274 PMC3605113

[ref16] Euesden, J., Lewis, C. M., & O'Reilly, P. F. (2014). PRSice: Polygenic risk score software. Bioinformatics (Oxford, England), 31, 1466–1468. doi:10.1093/bioinformatics/btu848. PMC4410663 25550326

[ref17] Gao, Q., Xu, Q., Guo, X., Fan, H., & Zhu, H. (2017). Particulate matter air pollution associated with hospital admissions for mental disorders: A time-series study in Beijing, China. European Psychiatry, 44, 68–75. doi:10.1016/j.eurpsy.2017.02.492. 28545011

[ref18] Genomes Project Consortium (2015). A global reference for human genetic variation. Nature, 526, 68. doi:10.1038/nature15393. 26432245 PMC4750478

[ref19] Ho, B.-C., Nopoulos, P., Flaum, M., Arndt, S., & Andreasen, N. C. (1998). Two-year outcome in first-episode schizophrenia: Predictive value of symptoms for quality of life. American Journal of Psychiatry, 155, 1196–1201. doi:10.1176/ajp.155.9.1196. 9734542

[ref20] Horsdal, H. T., Agerbo, E., McGrath, J. J., Vilhjálmsson, B. J., Antonsen, S., Closter, A. M., … Webb, R. T. (2019). Association of childhood exposure to nitrogen dioxide and polygenic risk score for schizophrenia with the risk of developing schizophrenia. JAMA Network Open, 2, e1914401–e1914401. doi:10.1001/jamanetworkopen.2019.14401. 31675084 PMC6827271

[ref21] Howes, O. D., & Murray, R. M. (2014). Schizophrenia: An integrated sociodevelopmental-cognitive model. The Lancet, 383, 1677–1687. doi:10.1016/S0140-6736(13)62036-X. PMC4127444 24315522

[ref22] Howie, B. N., Donnelly, P., & Marchini, J. (2009). A flexible and accurate genotype imputation method for the next generation of genome-wide association studies. Plos Genetics, 5, e1000529. doi:10.1371/journal.pgen.1000529. 19543373 PMC2689936

[ref23] Kirkbride, J. B., Jones, P. B., Ullrich, S., & Coid, J. W. (2014). Social deprivation, inequality, and the neighborhood-level incidence of psychotic syndromes in East London. Schizophrenia Bulletin, 40, 169–180. doi:10.1093/schbul/sbs151. 23236081 PMC3885290

[ref24] Lederbogen, F., Kirsch, P., Haddad, L., Streit, F., Tost, H., Schuch, P., … Deuschle, M. (2011). City living and urban upbringing affect neural social stress processing in humans. Nature, 474, 498–501. doi:10.1038/nature10190. 21697947

[ref25] Legge, S. E., Jones, H. J., Kendall, K. M., Pardiñas, A. F., Menzies, G., Bracher-Smith, M., … Hotopf, M. (2019). Association of genetic liability to psychotic experiences with neuropsychotic disorders and traits. JAMA Psychiatry, 76, 1256–1265. doi:10.1001/jamapsychiatry.2019.2508. 31553412 PMC6764002

[ref26] Lichtenstein, P., Yip, B. H., Björk, C., Pawitan, Y., Cannon, T. D., Sullivan, P. F., & Hultman, C. M. (2009). Common genetic determinants of schizophrenia and bipolar disorder in Swedish families: A population-based study. The Lancet, 373, 234–239. doi:10.1016/S0140-6736(09)60072-6. PMC3879718 19150704

[ref27] Loewy, R. L., Pearson, R., Vinogradov, S., Bearden, C. E., & Cannon, T. D. (2011). Psychosis risk screening with the Prodromal Questionnaire—brief version (PQ-B). Schizophrenia Research, 129, 42–46. doi:10.1016/j.schres.2011.03.029. 21511440 PMC3113633

[ref28] Martin, A. R., Gignoux, C. R., Walters, R. K., Wojcik, G. L., Neale, B. M., Gravel, S., … Kenny, E. E. (2017). Human demographic history impacts genetic risk prediction across diverse populations. The American Journal of Human Genetics, 100, 635–649. doi:10.1016/j.ajhg.2017.03.004. 28366442 PMC5384097

[ref29] Milne, B., Moffitt, T., Crump, R., Poulton, R., Rutter, M., Sears, M., … Caspi, A. (2008). How should we construct psychiatric family history scores? A comparison of alternative approaches from the Dunedin Family Health History Study. Psychological Medicine, 38, 1793–1802. doi:10.1017/S0033291708003115. 18366822 PMC3752774

[ref30] Moffitt T. E. and The E-Risk Study Team (2002). Teen-aged mothers in contemporary Britain. Journal of Child Psychology and Psychiatry and Allied Disciplines, 43, 727–742. doi:10.1111/1469-7610.00082. 12236608

[ref31] Mortensen, P. B., Pedersen, C. B., Westergaard, T., Wohlfahrt, J., Ewald, H., Mors, O., … Melbye, M. (1999). Effects of family history and place and season of birth on the risk of schizophrenia. New England Journal of Medicine, 340, 603–608. doi:10.1056/nejm199902253400803. 10029644

[ref32] Newbury, J. B., Arseneault, L., Beevers, S. D., Kitwiroon, N., Roberts, S., Pariante, C. M., … Fisher, H. L. (2019). Association of air pollution exposure with psychotic experiences during adolescence. JAMA Psychiatry, 76, 614–623. doi:10.1001/jamapsychiatry.2019.0056. 30916743 PMC6499472

[ref33] Newbury, J. B., Arseneault, L., Caspi, A., Moffitt, T. E., Odgers, C. L., Baldwin, J. R., … Fisher, H. L. (2017). In the eye of the beholder: Perceptions of neighborhood adversity and psychotic experiences in adolescence. Development and Psychopathology, 29, 1823–1837. doi:10.1017/S0954579417001420. 29162184 PMC5912687

[ref34] Newbury, J., Arseneault, L., Caspi, A., Moffitt, T. E., Odgers, C. L., & Fisher, H. L. (2016). Why are children in urban neighborhoods at increased risk for psychotic symptoms? Findings from a UK longitudinal cohort study. Schizophrenia Bulletin, 42, 1372–1383. doi:10.1093/schbul/sbw052. 27153864 PMC5049530

[ref35] Newbury, J., Arseneault, L., Caspi, A., Moffitt, T. E., Odgers, C. L., & Fisher, H. L. (2018). Cumulative effects of neighborhood social adversity and personal crime victimization on adolescent psychotic experiences. Schizophrenia Bulletin, 44, 348–358. doi:10.1093/schbul/sbx060. 28535284 PMC5815129

[ref36] Odgers, C. L., Caspi, A., Bates, C. J., Sampson, R. J., & Moffitt, T. E. (2012 *a*). Systematic social observation of children's neighborhoods using Google Street View: A reliable and cost-effective method. Journal of Child Psychology and Psychiatry and Allied Disciplines, 53, 1009–1017. doi:10.1111/j.1469-7610.2012.02565.x. 22676812 PMC3537178

[ref37] Odgers, C. L., Caspi, A., Russell, M. A., Sampson, R. J., Arseneault, L., & Moffitt, T. E. (2012 *b*). Supportive parenting mediates neighborhood socioeconomic disparities in children's antisocial behavior from ages 5 to 12. Development and Psychopathology, 24, 705–721. doi:10.1017/S0954579412000326. 22781850 PMC3551477

[ref38] Odgers, C. L., Moffitt, T. E., Tach, L. M., Sampson, R. J., Taylor, A., Matthews, C. L., & Caspi, A. (2009). The protective effects of neighborhood collective efficacy on British children growing up in deprivation: A developmental analysis. Developmental Psychology, 45, 942–957. doi:10.1037/a0016162. 19586172 PMC4212822

[ref39] O'Donoghue, B., Roche, E., & Lane, A. (2016). Neighbourhood level social deprivation and the risk of psychotic disorders: A systematic review. Social Psychiatry and Psychiatric Epidemiology, 51, 941–950. doi:10.1007/s00127-016-1233-4. 27178430

[ref40] Office for National Statistics (2013). Urban and rural area definitions for policy purposes in England and Wales: Methodology (v1.0). Retrieved from https://assets.publishing.service.gov.uk/government/uploads/system/uploads/attachment_data/file/239477/RUC11methodologypaperaug_28_Aug.pdf.

[ref41] Pain, O., Dudbridge, F., Cardno, A. G., Freeman, D., Lu, Y., Lundstrom, S., … Ronald, A. (2018). Genome-wide analysis of adolescent psychotic-like experiences shows genetic overlap with psychiatric disorders. American Journal of Medical Genetics Part B: Neuropsychiatric Genetics, 177, 416–425. doi:10.1002/ajmg.b.32630. PMC6001485 29603866

[ref42] Paksarian, D., Trabjerg, B., Merikangas, K., Mors, O., Børglum, A., Hougaard, D., … Agerbo, E. (2018). The role of genetic liability in the association of urbanicity at birth and during upbringing with schizophrenia in Denmark. Psychological Medicine, 48, 305–314. doi:10.1017/S0033291717001696. 28659227 PMC6361630

[ref43] Pardiñas, A. F., Holmans, P., Pocklington, A. J., Escott-Price, V., Ripke, S., Carrera, N., … Hamshere, M. L. (2018). Common schizophrenia alleles are enriched in mutation-intolerant genes and in regions under strong background selection. Nature Genetics, 50, 381–389. doi:10.1038/s41588-018-0059-2. 29483656 PMC5918692

[ref44] Pedersen, C. B. (2001). Family history, place and season of birth as risk factors for schizophrenia in Denmark: A replication and reanalysis. The British Journal of Psychiatry, 179, 46–52. doi:10.1192/bjp.179.1.46. 11435268

[ref45] Pedersen, C. B., & Mortensen, P. B. (2001). Evidence of a dose-response relationship between urbanicity during upbringing and schizophrenia risk. Archives of General Psychiatry, 58, 1039–1046. doi:10.1001/archpsyc.58.11.1039. 11695950

[ref46] Plomin, R., Haworth, C. M. A., & Davis, O. S. P. (2011). Common disorders are quantitative traits. Nature Reviews: Genetics, 10, 872. doi:10.1038/nrg2670. 19859063

[ref47] Polanczyk, G., Moffitt, T. E., Arseneault, L., Cannon, M., Ambler, A., Keefe, S. E. R., … Caspi, A. (2010). Etiological and clinical features of childhood psychotic symptoms: Results from a birth cohort. Archives of General Psychiatry, 67, 328–338. doi:10.1001/archgenpsychiatry.2010.14. 20368509 PMC3776482

[ref48] Poulton, R., Caspi, A., Moffitt, T. E., Cannon, M., Murray, R. M., & Harrington, H. (2000). Children's self-reported psychotic symptoms and adult schizophreniform disorder: A 15-year longitudinal study. Archives of General Psychiatry, 57, 1053–1058. doi:10.1001/archpsyc.57.11.1053. 11074871

[ref49] Ripke, S., Neale, B. M., Corvin, A., Walters, J. T., Farh, K.-H., Holmans, P. A., … Huang, H. (2014). Biological insights from 108 schizophrenia-associated genetic loci. Nature, 511, 421. doi:10.1038/nature13595. 25056061 PMC4112379

[ref50] Robins, L., Cottler, L., Bucholz, K., & Compton, W. (1995). Diagnostic interview schedule for DSM-IV *(*DIS-IV*)*. St Louis, MO: Washington University School of Medicine.

[ref51] Rogers, W. (1994). Regression standard errors in clustered samples. Stata Technical Bulletin, 3, 19–23. doi:RePEc:tsj:stbull:y:1994:v:3:i:13:sg17.

[ref52] Sariaslan, A., Fazel, S., D'Onofrio, B., Långström, N., Larsson, H., Bergen, S., … Lichtenstein, P. (2016). Schizophrenia and subsequent neighborhood deprivation: Revisiting the social drift hypothesis using population, twin and molecular genetic data. Translational Psychiatry, 6, e796. doi:10.1038/tp.2016.62. 27138795 PMC5070045

[ref53] Sherry, S. T., Ward, M.-H., Kholodov, M., Baker, J., Phan, L., Smigielski, E. M., & Sirotkin, K. (2001). dbSNP: The NCBI database of genetic variation. Nucleic Acids Research, 29, 308–311. doi:10.1093/nar/29.1.308. 11125122 PMC29783

[ref54] Solmi, F., Lewis, G., Zammit, S., & Kirkbride, J. (2019). Neighbourhood characteristics at birth and positive and negative psychotic symptoms in adolescence: Findings from the ALSPAC birth cohort. Schizophrenia Bulletin, 46, 581–591. doi:10.1093/schbul/sbz049. PMC7147568 31167032

[ref55] Spauwen, J., Krabbendam, L., Lieb, R., Wittchen, H. U., & van Os, J. (2004). Does urbanicity shift the population expression of psychosis? Journal of Psychiatric Research, 38, 613–618. doi:10.1016/j.jpsychires.2004.04.003. 15458857

[ref56] Stahl, S., & Buckley, P. F. (2007). Negative symptoms of schizophrenia: A problem that will not go away. Acta Psychiatrica Scandinavica, 115, 4–11. 17201860 10.1111/j.1600-0447.2006.00947.x

[ref57] Trzesniewski, K. H., Moffitt, T. E., Caspi, A., Taylor, A., & Maughan, B. (2006). Revisiting the association between reading achievement and antisocial behavior: New evidence of an environmental explanation from a twin study. Child Development, 77, 72–88. doi:10.1111/j.1467-8624.2006.00857.x. 16460526

[ref58] van Os, J., Linscott, R. J., Myin-Germeys, I., Delespaul, P., & Krabbendam, L. (2009). A systematic review and meta-analysis of the psychosis continuum: Evidence for a psychosis proneness-persistence-impairment model of psychotic disorder. Psychological Medicine, 39, 179–195. doi:10.1017/S0033291708003814. 18606047

[ref59] van Winkel, R., Stefanis, N. C., & Myin-Germeys, I. (2008). Psychosocial stress and psychosis. A review of the neurobiological mechanisms and the evidence for gene-stress interaction. Schizophrenia Bulletin, 34, 1095–1105. doi:10.1093/schbul/sbn101. 18718885 PMC2632486

[ref60] Vassos, E., Di Forti, M., Coleman, J., Iyegbe, C., Prata, D., Euesden, J., … Patel, H. (2017). An examination of polygenic score risk prediction in individuals with first-episode psychosis. Biological Psychiatry, 81, 470–477. doi:10.1016/j.biopsych.2016.06.028. 27765268

[ref61] Vassos, E., Pedersen, C. B., Murray, R. M., Collier, D. A., & Lewis, C. M. (2012). Meta-analysis of the association of urbanicity with schizophrenia. Schizophrenia Bulletin, 38, 1118–1123. doi:10.1093/schbul/sbs096. 23015685 PMC3494055

[ref62] Visscher, P. M., Wray, N. R., Zhang, Q., Sklar, P., McCarthy, M. I., Brown, M. A., & Yang, J. (2017). 10 Years of GWAS discovery: Biology, function, and translation. American Journal of Human Genetics, 101, 5–22. doi:10.1016/j.ajhg.2017.06.005. 28686856 PMC5501872

[ref63] Weissman, M. M., Wickramaratne, P., Adams, P., Wolk, S., Verdeli, H., & Olfson, M. (2000). Brief screening for family psychiatric history: The family history screen. Archives of General Psychiatry, 57, 675–682. doi:10-1001/pubs.Arch Gen Psychiatry-ISSN-0003-990x-57-7-yoa8214. 10891038 10.1001/archpsyc.57.7.675

[ref64] Wray N. R., Ripke S., Mattheisen M., Trzaskowski M., Byrne E. M., & Abdellaoui A., … the Major Depressive Disorder Working Group of the Psychiatric Genomics Consortium (2018). Genome-wide association analyses identify 44 risk variants and refine the genetic architecture of major depression. Nature Genetics, 50, 668–681. doi:10.1038/s41588-018-0090-3. 29700475 PMC5934326

[ref65] Yang, J., Visscher, P. M., & Wray, N. R. (2010). Sporadic cases are the norm for complex disease. European Journal of Human Genetics, 18, 1039–1043. doi:10.1038/ejhg.2009.177. 19826454 PMC2987426

[ref66] Yoshizumi, T., Murase, S., Honjo, S., Kaneko, H., & Murakami, T. (2004). Hallucinatory experiences in a community sample of Japanese children. Journal of the American Academy of Child and Adolescent Psychiatry, 43, 1030–1036. doi:10.1097/01.chi.0000126937.44875.6b. 15266199

[ref67] Yung, A. R., Nelson, B., Baker, K., Buckby, J. A., Baksheev, G., & Cosgrave, E. M. (2009). Psychotic-like experiences in a community sample of adolescents: Implications for the continuum model of psychosis and prediction of schizophrenia. Australian and New Zealand Journal of Psychiatry, 43, 118–128. doi:10.1080/00048670802607188. 19153919

