# Supplementary material for: Association between genetic and socioenvironmental risk for schizophrenia during upbringing in a UK longitudinal cohort
Source: Psychol Med. 2020 Sep 25;52(8):1527–37. doi: 10.1017/S0033291720003347 (PMC9226384; doi:10.1017/S0033291720003347)
Supplement: Supplementary file 1 [file S0033291720003347sup001.docx]

**Supplementary Materials**

***Newbury et al. Association between genetic and socioenvironmental risk for schizophrenia during upbringing in a UK longitudinal cohort.***

*Sample*

Participants were members of the Environmental Risk (E-Risk) Longitudinal Twin Study, which investigates how genetic and environmental factors shape children’s development. The sampling frame from which the E-Risk families were drawn was two consecutive birth cohorts (1994 and 1995) in a birth register of twins born in England and Wales (Trouton et al., 2002). Of the 15,906 twin pairs born in these two years, 71% joined the register. The E-Risk Study probability sample was drawn using a high-risk stratification strategy. High-risk families were those in which the mother had her first birth when she was 20 years of age or younger. This sampling frame was used (1) to replace high risk families who were selectively lost to the register via non-response and (2) to ensure sufficient base rates of environmental risk factors. Age at first childbearing was used as the risk-stratification variable because it was present for virtually all families in the register, it is relatively free of measurement error, and early childbearing is associated with a host of other difficulties and is a known risk factor for children’s problem behaviours (Maynard, 1997; Moffitt & The E-Risk Study Team, 2002). The high-risk sampling strategy resulted in a final sample in which one-third of Study mothers constitute a 160% oversample of mothers who were at high risk based on their young age at first birth (13–20 years), while the other two-thirds of Study mothers accurately represent all mothers in the general population (13–48 years) in England and Wales in 1994–95 (estimates derived from the General Household Survey; Bennett et al., 1996).

The Study sought a sample size of 1,100 families to allow for attrition in future years of the longitudinal study while retaining statistical power. An initial list of families who had same-sex twins was drawn from the register to target for home-visits, with a 10% oversample to allow for nonparticipation. Of the 1,203 families from the initial list who were eligible for inclusion, 1,116 (93%) participated in home-visit assessments when the twins were age 5 years, forming the base sample for the study (2,232 children): 4% of families refused, and 3% were lost to tracing or could not be reached after many attempts. With parent’s permission, questionnaires were posted to the children’s teachers, and teachers returned questionnaires for 94% of cohort children. Zygosity was determined using a standard zygosity questionnaire, which has been shown to have 95% accuracy (Price et al., 2000). Ambiguous cases were zygosity-typed using DNA. Subsequently, all participants who provided a DNA sample at any point across the study phases (97%) have been genotyped and had their zygosity checked. The sample includes 56% monozygotic (MZ) and 44% dizygotic (DZ) twin pairs. Sex is evenly distributed within zygosity (49% male). All families are English speaking, and the majority (93.7%) are White.

Attrition has been minimal, and data has been successfully collected from 98% (at age 7 years), 96% (at age 10 years), 96% (at age 12 years), and most recently in 2012–2014, 93% of the original sample (at age 18 years). Home-visits at ages 5, 7, 10, and 12 years included face-to-face assessments with participants as well as their mother (or primary caregiver); the home-visit at age 18 included interviews only with the participants, and questionnaires completed by co-informants (caregivers and other family members). Each twin participant was assessed by a different interviewer. Most participants (71.4%; N=1475) lived at the same address between ages 12 and 18. In addition, adolescents who did move house tended to move to similar neighborhoods: 87.0% of movers who lived in urban/intermediate neighborhoods at age 12 also lived in urban/intermediate neighborhoods at age 18.

The Joint South London and Maudsley and the Institute of Psychiatry Research Ethics Committee approved each phase of the study. Parents gave informed consent at ages 5–12. Participants gave assent at ages 5–12 and informed consent at age 18.

*Measures*

*Adolescent psychotic experiences.* To measure adolescent psychotic experiences, E-Risk families were visited by mental health trainees or professionals when children were aged 18. Interviewers had no prior knowledge about the participant. Each participant was privately interviewed about thirteen psychotic experiences they may have experienced since the age of 12, including seven items pertaining to hallucinations and delusions, such as: Have other people ever read your thoughts? Have you ever believed that you were being sent special messages through the television or radio, or that a programme has been arranged just for you alone? Have you ever thought you were being followed or spied on? Have you ever heard voices that other people cannot hear? Have you ever felt like you were under the control of some special power? Have you ever known what another person was thinking, like you could read their mind? Have you ever seen something or someone that other people could not see?; as well as six items about unusual feelings and thoughts such as: I have become more sensitive to lights or sounds, I feel as though I can’t trust anyone, I worry that my food may be poisoned, People or places I know seem different, I believe I have special abilities or powers beyond my natural talents, and My thinking is unusual or frightening. The item choice was guided by the Dunedin Study's age-11 interview protocol (Poulton et al., 2000), an instrument prepared for the Avon Longitudinal Study of Parents and Children (Schreier et al., 2009), and item pools since formalised in prodromal psychosis screening instruments including the Prevention through Risk Identification, Management and Education (PRIME)-screen (Miller et al., 2004), and the Structured Interview for Psychosis-Risk Syndromes (SIPS) (Miller et al., 2003). Interviewers coded each of the 13 items (7 hallucination/delusion items plus 6 unusual experiences items) 0, 1, 2, indicating respectively “not present”, “probably present” and “definitely present”. Responses to each of the 13 items (none, probable, definite) were summed to create a psychotic experiences scale (potential range=0–26, actual range=0–18, M=1.19, SD=2.58). The psychotic experiences measure did not involve clinical verification, meaning that this is a self-report measure capturing a broader range of mild, moderate and potentially clinically pertinent hallucinations, delusions, and other unusual feelings and thoughts. Since there were low numbers of adolescents with high psychotic experiences scores (e.g., only 1.0% [N=21] of participants had a psychotic experiences score of 13 or more), scores were placed into an ordinal scale to tackle the skewed distribution while retaining more information than a binary score. Just over 30% of participants had at least one psychotic experience between ages 12 and 18: 69.8% reported no psychotic experiences (coded 0; N=1,440), 15.5% reported 1 or 2 psychotic experiences (coded 1; N=319), 8.1% reported 3–5 psychotic experiences (coded 2: N=166), and 6.7% reported 6 or more psychotic experiences (coded 3: N=138). This 30.2% prevalence is similar to the prevalence of self-reported psychotic experiences in other community samples of teenagers and young adults (Spauwen et al., 2004; Yoshizumi et al., 2004; Yung et al., 2009).

*Environmental risks*

*Urbanicity*. Urbanicity was derived from the Office for National Statistics’ (ONS) Rural-Urban Definition for Small Area Geographies (RUC2011) classifications (Office for National Statistics, 2013). The ONS classifications utilised 2011 census data and were designed for application to small geostatistical units (e.g. Output Areas). Detailed information on how the ONS created the RUC2011 classifications of urbanicity is available on the ONS webpages (<https://www.gov.uk/government/uploads/system/uploads/attachment_data/file/239477/RUC11methodologypaperaug_28_Aug.pdf>). Briefly, RUC2011 was created by laying a grid of hectare cells (100m^2^) over England and Wales. Postcode addresses were assigned to cells, and residential densities were then calculated for increasing radii around each cell, providing each residential property with a density profile. This was combined with Output Area and contextual data such as settlement size and population sparsity, allowing each settlement to be assigned to one of ten urbanicity categories (Rural categories: sparse/non-sparse hamlets and isolated dwellings, sparse/non-sparse villages, sparse/non-sparse rural town and fringe; Urban categories: sparse/non-sparse city and town, and minor/major conurbations [conurbations are densely populated, large urban regions resulting from the expansion and coalescence of adjacent cities and towns]). ONS urbanicity scores were then assigned to every E-Risk family via the family’s postcode when children were aged 5, 12 and 18. Given the low numbers within some rural categories, urbanicity was collapsed into three levels (1: “rural” = all rural categories; 2: “intermediate” = urban cities and towns; and 3: “urban” = minor/major conurbations). E-Risk participants are nationally-representative in terms of ONS urbanicity classifications; for instance, the nationwide distribution of rural, intermediate, and urban residents in the UK is 18.9%, 45.0%, and 36.1%, respectively.

*Air pollution.* Pollution exposure estimates were modelled and linked to the latitude-longitude coordinates of participants’ home addresses at ages 10 and 18. Pollution estimates were modelled using CMAQ-urban, which is a coupled regional chemical transport model and street-scale dispersion model. CMAQ-urban uses a new generation of road traffic emissions inventory in the UK to model air quality down to individual streets, providing hourly estimates of pollutants at 20x20 metre grid points throughout the UK (i.e., address-level). Full details on the creation and validation of this model have been described previously (Beevers et al., 2012; Carslaw, 2011). The pollution estimates achieved good model performance against ground-based measurements (see Supplementary Table 4). The present study focusses on nitrogen dioxide (NO_2_), a regulated gaseous pollutant, which has previously been shown to be associated with adolescent psychotic experiences in this sample (Newbury et al., 2019). Genotyped participants’ exposure to NO_2_ was estimated by averaging hourly concentrations across the year (age 10/2004: M=25.14µg/m^3­­­^, SD=9.78, range=2.59-57.87; age 18/2012: M=.18.65µg/m^3^, SD=8.81, range=2.03-69.48). The present study categorizes NO_2_ into quartiles. Cut-offs for quartiles were 18.86µg/m^3­^, 24.74µg/m^3^, and 33.04µg/m^3^ at age 10; and 13.00µg/m^3^, 17.51µg/m^3^, and 25.05µg/m^3^ at age 18.

Neighborhood deprivation was constructed using A Classification of Residential Neighborhoods (ACORN), a geodemographic discriminator developed by CACI Information Services (<http://www.caci.co.uk/>) (CACI Information Services, 2006). Detailed information about ACORN’s classification of neighborhood-level socioeconomic-status (SES) has been provided previously (Caspi et al., 2000; Odgers et al., 2012b; Odgers et al., 2009). Briefly, CACI utilised over 400 variables from 2001 census data for Great Britain (e.g., educational qualifications, unemployment, housing tenure) and CACI’s consumer lifestyle database. Following hierarchical-cluster-analysis, five distinct and homogeneous ordinal groups were created ranging from “Wealthy Achiever” (coded 1) to “Hard Pressed” (coded 5) neighborhoods. Each family in our sample was matched to the ACORN code for its neighborhood via its postcode at ages 5, 12 and 18 (Caspi et al., 2000).

*Neighborhood crime.* Crime data in 2011 (the first year for which full street-level data was available), including information on the type of crime, date of occurrence, and approximate location, were accessed online as part of an open data sharing effort about crime and policing in England and Wales. Street-level crime data was extracted for each of the geospatial coordinates marking the family’s home (for a description see: <https://data.police.uk/about/#location-anonymisation>). Neighborhood crime rates were calculated by mapping a one-mile radius around each E-Risk Study participant’s home and tallying the total number of crimes that occurred in the area each month (for genotyped participants: M=214, SD=227, range=1–1781). These monthly crime rates were calculated for 2011, and then collapsed into quartiles. This measure covers various forms of crime, including violent offenses (e.g., assaults), sexual offenses (e.g., rape), robberies, burglaries, theft, arson, and vandalism.

*Neighborhood disorder and social cohesion.* Social conditions (i.e., social processes) were estimated via a postal survey sent in 2008 to residents living alongside E-Risk families (Odgers et al., 2012a; Odgers et al., 2009). In Britain, a postcode area typically contains 15 households, with at most 100 households (e.g., large apartment block). This type of postcode-level resolution represents a marked advantage over many existing neighborhood studies in which much larger census tract or census block units of analysis are used. Our objective was to obtain multiple reporters (e.g., 2 or more) for each family’s neighborhood (here defined to the street or apartment block level). Considering that the typical response rate for neighborhood surveys is approximately 30% (Messner et al., 2004), questionnaires were sent to every household in the same postcode as the E-Risk families, excluding the E-Risk families themselves (addresses were identified from electoral roll records). The number of surveys sent per postcode ranged from 15 to 50 residences per neighborhood (M=18.96, SE=0.21). Excluding undelivered surveys (N=600), the overall response rate was 28.1% (5601/19926), similar to that previously found (Messner et al., 2004). Survey respondents typically lived on the same street or within the same apartment block as the children in our study. Surveys were returned by an average of 5.18 (SD=2.73) respondents per neighborhood (range=0–18 respondents). There were at least three responses for 80% of neighborhoods and at least two responses from 95% of the neighborhoods (N=5,601 respondents) (Odgers et al., 2012a). Most respondents had lived in the neighborhood for more than 5 years (83%), and only 1% of respondents had lived in the neighborhood for less than 1 year. Neighborhood disorder (Sampson & Raudenbush, 1999) (14 items, each coded 0-2) was assessed by asking residents whether certain problems affected their neighborhood, including muggings, assaults, vandalism, graffiti and deliberate damage to property, etc. Social cohesion (Sampson et al., 1997) (5 items, each coded 0-4) was assessed by asking residents whether their neighbors shared values and trusted and got along with each other, etc. Items within each neighborhood characteristic scale were averaged to create summary scores from each of the 5601 resident respondents. Neighborhood characteristic scores for each E-Risk family were then created by averaging the summary scores of respondents within that family’s neighborhood. Among genotyped participants, the distribution of the social cohesion variable was M=2.24, SD=0.50, range=0-3.71; and of neighborhood disorder was M=0.47, SD=0.34, range=0-1.93). For the present study neighborhood disorder and social cohesion are categorized into tertiles at the 33^rd^ and 66^th^ centiles.

*Residential mobility*. Residential moves were measured at ages 5, 7, 10, 12 and 18 during private interviews. At ages 5, 7, 10, and 12, mothers reported on the number of residential moves their child had experienced since birth (or the previous visit) using life history calendars. At age 18 the participants themselves reported on whether they had moved to a different address since age 12. Residential moves were summed across phases 5–12 and phases 5–18, and collapsed into three categories including 1 (no moves), 2 (1 move), and 3 (2 or more moves).

*Family poverty*. Family socioeconomic status (SES) was measured when children were aged 5 via a composite of parental income, parental education, and parental occupation during interviews with the child’s primary caregiver (typically the mother): parental income was measured as the entire income of the household; parental education was the highest level of education achieved by either the mother or father (highest value taken), ranging from 1 (CSE [1], O Level [A-C], GCSE [A-C]) to 7 (postgraduate degree); parental occupation was the highest level of parental occupation of either parent, ranging from 1 (both parents unemployed [coded 2 if single unemployed mother]) to 9 (professional). The three SES indicators were highly correlated (r’s=0.57–0.67, all p’s<0.05) and loaded significantly onto one latent factor (M=2.00, SD= 0.82; factor loadings=0.80, 0.70 and 0.83 for

parental income, education and occupation, respectively). These variables were then standardised and summed, before categorising into tertiles at the 33.33rd and 66.66^th^ centile (low-, medium-, and high-SES) (Trzesniewski et al., 2006). The present study uses a reverse-scored measure from 1 (high-SES) to 3 (low-SES).

*Multiple imputation using chained equations.* Multiple imputations were conducted for European ancestry, genotyped participants (N=1,999) for all analysis variables with missing values, including adolescent psychotic experiences, maternal psychotic symptoms, family psychiatric history, latent genetic risk, urbanicity, neighborhood deprivation, air pollution, neighborhood crime, neighborhood disorder, social cohesion, and residential mobility. Multiple imputations were conducted using chained equations in Stata v16.0 (“mi impute chained”) because this method is suitable for arbitrary missing-value patterns (e.g., where participants are missing data for one phase but not a subsequent phase or where participants are missing data for one variable at one phase but not another variable for the same phase). Missing values using chained equations are imputed iteratively through a sequence of univariate imputations with fully conditional specification of prediction equations. Prior to imputation, maternal psychotic symptoms, family psychiatric history, and latent genetic risk were specified as continuous with a restricted range using “truncreg”, and all other variables were specified as ordinal using “ologit”. We included some variables with complete data within models, including family ID (the family-level variable used to account for clustering of twins within families), schizophrenia PRS, depression PRS, sex, and family SES which all had no missing values in the target dataset (N=1,999). We imputed 10 datasets using a random seed of 1234. Between N=65 (neighborhood disorder and social cohesion) and N=521 (environmental risk scale) values were imputed for analysis variables. Coefficients from the analysis of multiply imputed data were requested as odds ratios to enable comparison with complete case analyses.

| Participants (%) |  |
| --- | --- |
|  | Index of Multiple Deprivation Deciles  (1=most deprived, 10=least deprived) |

**Supplementary Figure 1.** Distribution of E-Risk participants at age 18 across Index of Multiple Deprivation deciles

**Supplementary Table 1.** Association of socioenvironmental risk factors with adolescent psychotic experiences, following multiple imputations.

| **Socioenvironmental risk factors** | **Level** | **Model adjustment** | | | | | | |
| --- | --- | --- | --- | --- | --- | --- | --- | --- |
|  |  | **Unadjusted** | **Schizophrenia PRS** | **Depression PRS** | **Maternal psychotic symptoms** | **Family psychiatric history** | **Latent genetic risk** | **All genetic risk indices together** |
|  |  | **OR**  **(95% CI)** | **OR**  **(95% CI)** | **OR**  **(95% CI)** | **OR**  **(95% CI)** | **OR**  **(95% CI)** | **OR**  **(95% CI)** | **OR**  **(95% CI)** |
|  |  |  |  |  |  |  |  |  |
| Environmental risk scale | 0 | Ref | Ref | Ref | Ref | Ref | Ref | Ref |
|  | 1 | 1.54*  (1.08 – 2.21) | 1.54*  (1.07 – 2.20) | 1.54*  (1.08 – 2.22) | 1.56*  (1.09 – 2.24) | 1.48*  (1.03 – 2.12) | 1.47*  (1.05 – 2.05) | 1.42*  (1.01 – 1.99) |
|  | 2 | 1.62*  (1.12 – 2.34) | 1.62*  (1.11 – 2.34) | 1.60*  (1.10 – 2.32) | 1.63*  (1.13 – 2.35) | 1.59*  (1.10 – 2.29) | 1.57**  (1.13 – 2.18) | 1.52*  (1.09 – 2.12) |
|  | 3 | 1.77**  (1.19 – 2.63) | 1.73**  (1.16 – 2.59) | 1.72**  (1.16 – 2.57) | 1.77**  (1.19 – 2.63) | 1.68*  (1.13 – 2.51) | 1.65**  (1.16 – 2.35) | 1.55*  (1.09 – 2.21) |
|  | 4 | 2.67***  (1.73 – 4.13) | 2.62***  (1.69 – 4.07) | 2.60***  (1.67 – 4.05) | 2.63***  (1.70 – 4.06) | 2.52***  (1.63 – 3.90) | 2.34***  (1.56 – 3.50) | 2.17***  (1.43 – 3.28) |
|  | 5+ | 2.98***  (1.88 – 4.73) | 2.93***  (1.85 – 4.64) | 2.88***  (2.81 – 4.58) | 2.91***  (1.84 – 4.62) | 2.70***  (1.71 – 4.25) | 2.64***  (1.76 – 3.98) | 2.34***  (1.55 – 3.54) |
|  |  |  |  |  |  |  |  |  |
| Urbanicity (age 5) | 1 | Ref | Ref | Ref | Ref | Ref | Ref | Ref |
|  | 2 | 1.26  (0.92 – 1.72) | 1.24  (0.91 – 2.04) | 1.24  (0.91 – 1.70) | 1.25  (0.92 – 1.72) | 1.21  (0.88 – 1.65) | 1.18  (0.89 – 1.57) | 1.14  (0.86 – 1.52) |
|  | 3 | 1.49*  (1.06 – 2.08) | 1.46*  (1.04 – 2.04) | 1.47*  (1.05 – 2.05) | 1.50*  (1.07 – 2.11) | 1.46*  (1.04 – 2.04) | 1.43*  (1.06 – 1.92) | 1.39*  (1.03 – 1.87) |
|  |  |  |  |  |  |  |  |  |
| Urbanicity (age 12) | 1 | Ref | Ref | Ref | Ref | Ref | Ref | Ref |
|  | 2 | 1.33†  (0.98 – 1.80) | 1.31†  (0.97 – 1.78) | 1.31†  (0.97 – 1.78) | 1.34†  (0.99 – 1.81) | 1.31†  (0.97 – 1.77) | 1.22  (0.93 – 1.58) | 1.20  (0.92 – 1.57) |
|  | 3 | 1.51*  (1.09 – 2.09) | 1.48*  (1.07 – 2.05) | 1.50*  (1.08 – 2.08) | 1.52*  (1.10 – 2.11) | 1.51*  (1.10 – 2.09) | 1.43*  (1.08 – 1.90) | 1.42*  (1.06 – 1.90) |
|  |  |  |  |  |  |  |  |  |
| Air pollution | 1 | Ref | Ref | Ref | Ref | Ref | Ref | Ref |
|  | 2 | 0.76†  (0.56 – 1.04) | 0.76†  (0.56 – 1.05) | 0.76†  (0.55 – 1.04) | 0.75†  (0.55 – 1.02) | 0.78  (0.57 – 1.07) | 0.79†  (0.60 – 1.03) | 0.80  (0.61 – 1.06) |
|  | 3 | 0.99  (0.73 – 1.35) | 0.99  (0.73 – 1.35) | 1.00  (0.73 – 1.37) | 0.98  (0.72 – 1.34) | 1.00  (0.73 – 1.37) | 0.99  (0.76 – 1.30) | 1.01  (0.76 – 1.33) |
|  | 4 | 1.43*  (1.06 – 1.93) | 1.41*  (1.04 – 1.91) | 1.40*  (1.04 – 1.89) | 1.40*  (1.04 – 1.90) | 1.40*  (1.04 – 1.90) | 1.33*  (1.02 – 1.72) | 1.27†  (0.98 – 1.66) |
|  |  |  |  |  |  |  |  |  |
| Neighborhood deprivation (age 5) | 1 | Ref | Ref | Ref | Ref | Ref | Ref | Ref |
|  | 2 | 1.46  (0.78 – 2.74) | 1.44  (0.77 – 2.71) | 1.43  (0.77 – 2.68) | 1.49  (0.80 – 2.77) | 1.41  (0.77 – 2.60) | 1.34  (0.77 – 2.32) | 1.27  (0.73 – 2.19) |
|  | 3 | 1.33†  (0.95 – 1.85) | 1.33†  (0.95 – 1.85) | 1.35†  (0.97 – 1.88) | 1.33†  (0.95 – 1.85) | 1.32  (0.95 – 1.83) | 1.24  (0.93 – 1.65) | 1.25  (0.93 – 1.67) |
|  | 4 | 1.70**  (1.16 – 2.47) | 1.69**  (1.16 – 2.46) | 1.66**  (1.13 – 2.42) | 1.67**  (1.14 – 2.44) | 1.67**  (1.14 – 2.45) | 1.55**  (1.12 – 2.14) | 1.51*  (1.09 – 2.09) |
|  | 5 | 1.87***  (1.38 – 2.55) | 1.85***  (1.36 – 2.52) | 1.85***  (1.36 – 2.53) | 1.85***  (1.36 – 2.52) | 1.81***  (1.33 – 2.47) | 1.75***  (1.34 – 2.30) | 1.69***  (1.29 – 2.23) |
|  |  |  |  |  |  |  |  |  |
| Neighborhood deprivation (age 12) | 1 | Ref | Ref | Ref | Ref | Ref | Ref | Ref |
|  | 2 | 1.90*  (1.06 – 3.41) | 1.82*  (1.01 – 3.27) | 1.81*  (1.01 – 3.25) | 1.92*  (1.07 – 3.43) | 1.82*  (1.02 – 3.23) | 1.62†  (0.95 – 2.77) | 1.59  (0.86 – 2.55) |
|  | 3 | 1.54**  (1.13 – 2.10) | 1.54**  (1.13 – 2.10) | 1.54**  (1.13 – 2.10) | 1.54**  (1.13 – 2.10) | 1.49*  (1.09 – 2.04) | 1.47**  (1.13 – 1.92) | 1.45**  (1.11 – 1.90) |
|  | 4 | 1.62*  (1.11 – 2.35) | 1.58*  (1.08 – 2.90) | 1.59*  (1.09 – 2.31) | 1.56*  (1.08 – 2.27) | 1.54*  (1.05 – 2.26) | 1.54*  (1.11 – 2.13) | 1.44*  (1.03 – 2.02) |
|  | 5 | 2.14***  (1.56 – 2.93) | 2.11***  (1.54 – 2.90) | 2.09***  (1.53 – 2.87) | 2.09***  (1.53 – 2.87) | 2.04***  (1.49 – 2.78) | 2.02***  (1.54 – 2.66) | 1.89***  (1.43 – 2.50) |
|  |  |  |  |  |  |  |  |  |
| Neighborhood disorder | 1 | Ref | Ref | Ref | Ref | Ref | Ref | Ref |
|  | 2 | 1.34*  (1.02 – 1.75) | 1.33*  (1.01 – 1.76) | 1.35*  (1.02 – 1.78) | 1.34*  (1.02 – 1.76) | 1.35*  (1.02 – 1.78) | 1.31*  (1.03 – 1.66) | 1.34*  (1.05 – 1.71) |
|  | 3 | 1.64**  (1.24 – 2.18) | 1.61**  (1.21 – 2.14) | 1.61**  (1.21 – 2.13) | 1.62**  (1.23 – 2.15) | 1.57**  (1.19 – 2.08) | 1.56**  (1.21 – 2.01) | 1.47**  (1.14 – 1.90) |
|  |  |  |  |  |  |  |  |  |
| Neighborhood social cohesion | 1 | Ref | Ref | Ref | Ref | Ref | Ref | Ref |
|  | 2 | 1.01  (0.77 – 1.32) | 1.00  (0.76 – 1.31) | 1.00  (0.77 – 1.31) | 1.01  (0.77 – 1.32) | 1.00  (0.77 – 1.32) | 1.01  (0.79 – 1.28) | 0.99  (0.77 – 1.26) |
|  | 3 | 1.55**  (1.19 – 2.02) | 1.53**  (1.18 – 1.99) | 1.50**  (1.15 – 1.96) | 1.55**  (1.19 – 2.01) | 1.51**  (1.16 – 1.97) | 1.49***  (1.19 – 1.86) | 1.42**  (1.13 – 1.79) |
|  |  |  |  |  |  |  |  |  |
| Residential mobility | 1 | Ref | Ref | Ref | Ref | Ref | Ref | Ref |
|  | 2 | 1.11  (0.85 – 1.46) | 1.00  (0.84 – 1.45) | 1.11  (0.85 – 1.47) | 1.09  (0.83 – 1.43) | 1.05  (0.80 – 1.38) | 1.09  (0.86 – 1.38) | 1.04  (0.82 – 1.33) |
|  | 3 | 1.31*  (1.00 – 1.71) | 1.28†  (0.98 – 1.68) | 1.27†  (0.97 – 1.67) | 1.27†  (0.97 – 1.67) | 1.16  (0.88 – 1.54) | 1.21†  (0.97 – 1.53) | 1.07  (0.85 – 1.36) |
|  |  |  |  |  |  |  |  |  |
| Family poverty | 1 | Ref | Ref | Ref | Ref | Ref | Ref | Ref |
|  | 2 | 1.66***  (1.25 – 2.20) | 1.65**  (1.25 – 2.19) | 1.65**  (1.24 – 2.18) | 1.64**  (1.24 – 2.18) | 1.64**  (1.24 – 2.18) | 1.59***  (1.25 – 2.03) | 1.56***  (1.22 – 2.00) |
|  | 3 | 2.17***  (1.65 – 2.85) | 2.14***  (1.63 – 2.82) | 2.13***  (1.62 – 2.80) | 2.10***  (1.60 – 2.77) | 2.01***  (1.52 – 2.65) | 2.02***  (1.60 – 2.55) | 1.85***  (1.45 – 2.35) |

Note: CI, confidence intervals; OR, odds ratio from ordinal logistic regression; Ref, reference category; † p<0.1; * p<0.05; ** p<0.01; *** p<0.001. ^a^ Levels of environmental risks were: Urbanicity, 1-3=rural to urban; Air pollution, 1-4=lowest to highest quartile; Neighborhood deprivation, 1-5=lowest to highest quintile; Neighborhood disorder, 1-3=lowest to highest tertile; Neighborhood social cohesion, 1-3=highest to lowest tertile; Residential mobility, 1-3=0, 1, 2+ residential moves; Family poverty, 1-3=high to low socio-economic status. Aside from the unadjusted model, all models control for sex in addition to the specified genetic risk measure. Missing values were multiply imputed using chained equations for genotyped, European ancestry participants (N=1,999). All analyses control for the non-independence of twin observations.

**Supplementary Table 2.** Association of genetic risk indices with socioenvironmental risk factors for schizophrenia – complete case analysis

| **Socioenvironmental risk factors** | **Genetic risk indices** | | | | |
| --- | --- | --- | --- | --- | --- |
|  | **Schizophrenia PRS** | **Depression PRS** | **Maternal psychotic symptoms** | **Family psychiatric history** | **Latent genetic risk** |
|  | **OR**  **(95% CI)** | **OR**  **(95% CI)** | **OR**  **(95% CI)** | **OR**  **(95% CI)** | **OR**  **(95% CI)** |
|  |  |  |  |  |  |
| Environmental risk scale (age 5-18) | 1.23**  (1.08 – 1.40) | 1.16*  (1.04 – 1.30) | 1.10  (0.95 – 1.28) | 1.26***  (1.10 – 1.44) | 1.18*  (1.04 – 1.33) |
| Urbanicity |  |  |  |  |  |
| Age 5 | 1.10  (0.98 – 1.23) | 1.06  (0.94 – 1.20) | 0.91†  (0.81 – 1.01) | 1.08  (0.95 – 1.22) | 1.10  (0.97 – 1.24) |
| Age 12 | 1.11†  (0.99 – 1.25) | 1.03  (0.91 – 1.16) | 0.92  (0.82 – 1.04) | 1.03  (0.91 – 1.17) | 1.13*  (1.01 – 1.27) |
| Age 18 | 1.15*  (1.01 – 1.30) | 1.04  (0.92 – 1.18) | 0.94  (0.83 – 1.05) | 1.06  (0.92 – 1.21) | 1.11†  (0.99 – 1.26) |
| Air pollution |  |  |  |  |  |
| Age 10 | 1.07  (0.95 – 1.19) | 1.10†  (0.99 – 1.23) | 0.99  (0.90 – 1.10) | 1.09  (0.97 – 2.23) | 1.12†  (0.99 – 1.26) |
| Age 18 | 1.07  (0.95 – 1.21) | 1.13*  (1.01 – 1.26) | 0.99  (0.88 – 1.12) | 1.03  (0.91 – 1.17) | 1.12†  (0.99 – 1.25) |
| Neighborhood deprivation |  |  |  |  |  |
| Age 5 | 1.08  (0.97 – 1.20) | 1.09  (0.97 – 1.22) | 1.07  (0.95 – 1.21) | 1.07  (0.95 – 1.20) | 1.13*  (1.01 – 1.27) |
| Age 12 | 1.07  (0.97 – 1.19) | 1.10†  (0.99 – 1.23) | 1.14*  (1.01 – 1.28) | 1.16*  (1.03 – 1.30) | 1.10†  (0.99 – 1.23) |
| Age 18 | 1.00  (0.89 – 1.11) | 1.18**  (1.05 – 1.32) | 1.10†  (0.99 – 1.23) | 1.16*  (1.03 – 1.31) | 1.15*  (1.03 – 1.29) |
| Neighborhood crime (age 18) | 1.14*  (1.01 – 1.29) | 1.12*  (1.00 – 1.25) | 1.10  (0.97 – 1.24) | 1.10  (0.97 – 1.24) | 1.13*  (1.01 – 1.26) |
| Neighborhood disorder (age 12) | 1.17**  (1.04 – 1.31) | 1.11†  (0.99 – 1.24) | 1.02  (0.89 – 1.16) | 1.14*  (1.01 – 1.30) | 1.14*  (1.01 – 1.29 |
| Neighborhood social cohesion (age 12) | 1.10  (0.98 – 1.23) | 1.14*  (1.02 – 1.28) | 0.98  (0.87 – 1.10) | 1.10  (0.97 – 1.25) | 1.12†  (1.00 – 1.26) |
| Residential mobility |  |  |  |  |  |
| Ages 5-12 | 1.15*  (1.02 – 1.31) | 1.05  (0.92 – 1.19) | 1.19**  (1.05 – 1.35) | 1.37***  (1.20 – 1.57) | 1.12†  (0.99 – 1.27) |
| Ages 5-18 | 1.19**  (1.06 – 1.34) | 1.05  (0.94 – 1.19) | 1.26***  (1.13 – 1.40) | 1.36***  (1.20 – 1.55) | 1.17*  (1.03 – 1.31) |
| Family poverty (age 5) | 1.12*  (1.00 – 1.24) | 1.11†  (1.00 – 1.24) | 1.19**  (1.05 – 1.34) | 1.29***  (1.14 – 1.47) | 1.14*  (1.02 – 1.27) |

Note: CI, confidence intervals; OR, odds ratio; PRS, polygenic risk score; † p<0.1; * p<0.05; ** p<0.01; *** p<0.001. All analyses control for the non-independence of twin observations and included only one twin per MZ twin pair. Analyses were conducted on complete cases where participants had data on all genetic risk indices: N=1,075 to N=1,444.

**Supplementary Table 3.** Association of socioenvironmental risk factors with adolescent psychotic experiences – complete case analysis.

| **Socioenvironmental risk factors** | Level | **Model adjustment** | | | | | | |
| --- | --- | --- | --- | --- | --- | --- | --- | --- |
|  |  | **Unadjusted** | **Schizophrenia PRS** | **Depression PRS** | **Maternal psychotic symptoms** | **Family psychiatric history** | **Latent genetic risk** | **All genetic risk indices together** |
|  |  | **OR**  **(95% CI)** | **OR**  **(95% CI)** | **OR**  **(95% CI)** | **OR**  **(95% CI)** | **OR**  **(95% CI)** | **OR**  **(95% CI)** | **OR**  **(95% CI)** |
|  |  |  |  |  |  |  |  |  |
| Environmental risk scale | 0 | Ref | Ref | Ref | Ref | Ref | Ref | Ref |
|  | 1 | 1.74**  (1.20 – 2.52) | 1.72**  (1.18 – 2.50) | 1.75**  (1.20 – 2.54) | 1.75**  (1.21 – 2.55) | 1.66**  (1.14 – 2.41) | 1.63**  (1.19 – 2.24) | 1.59**  (1.16 – 2.19) |
|  | 2 | 1.75**  (1.19 – 2.58) | 1.74**  (1.18 – 2.57) | 1.73**  (1.17 – 2.55) | 1.75**  (1.18 – 2.57) | 1.71**  (1.16 – 2.53) | 1.69**  (1.22 – 2.35) | 1.65**  (1.18 – 2.30) |
|  | 3 | 1.90**  (1.26 – 1.87) | 1.83**  (1.21 – 2.76) | 1.86**  (1.23 – 2.80) | 1.90**  (1.26 – 2.86) | 1.80**  (1.18 – 2.71) | 1.77**  (1.25 – 2.50) | 1.66**  (1.17 – 2.37) |
|  | 4 | 2.76***  (1.85 – 4.14) | 2.69***  (1.80 – 4.03) | 2.71***  (1.81 – 4.08) | 2.69***  (1.81 – 4.02) | 2.59***  (1.73 – 3.87) | 2.41***  (1.71 – 3.40) | 2.22***  (1.56 – 3.17) |
|  | 5+ | 3.13***  (1.94 – 5.05) | 3.05***  (1.89 – 4.90) | 3.05***  (1.89 – 4.93) | 3.00***  (1.86 – 4.83) | 2.80***  (1.75 – 4.48) | 2.71***  (1.81 – 4.05) | 2.39***  (1.59 – 3.60) |
|  |  |  |  |  |  |  |  |  |
| Urbanicity (age 5) | 1 | Ref | Ref | Ref | Ref | Ref | Ref | Ref |
|  | 2 | 1.12  (0.81 – 1.55) | 1.10  (0.80 – 1.53) | 1.11  (0.80 – 1.53) | 1.12  (0.81 – 1.55) | 1.07  (0.77 – 1.48) | 1.09  (0.83 – 1.43) | 1.03  (0.78 – 1.37) |
|  | 3 | 1.46*  (1.03 – 2.07) | 1.43*  (1.00 – 2.03) | 1.45*  (1.02 – 2.06) | 1.47*  (1.04 – 2.09) | 1.42*  (1.00 – 2.01) | 1.41*  (1.05 – 1.89) | 1.35†  (1.00 – 1.82) |
|  |  |  |  |  |  |  |  |  |
| Urbanicity (age 12) | 1 | Ref | Ref | Ref | Ref | Ref | Ref | Ref |
|  | 2 | 1.29  (0.94 – 1.76) | 1.27  (0.92 – 1.74) | 1.27  (0.92 – 1.74) | 1.29  (0.94 – 1.77) | 1.26  (0.92 – 1.73) | 1.19  (0.91 – 1.56) | 1.16  (0.89 – 1.53) |
|  | 3 | 1.52*  (1.08 – 2.15) | 1.49*  (1.05 – 2.11) | 1.52*  (1.07 – 2.15) | 1.54*  (1.09 – 2.18) | 1.52*  (1.08 – 2.15) | 1.43*  (1.07 – 1.93) | 1.42*  (1.05 – 1.92) |
|  |  |  |  |  |  |  |  |  |
| Air pollution | 1 | Ref | Ref | Ref | Ref | Ref | Ref | Ref |
|  | 2 | 0.75†  (0.54 – 1.05) | 0.75†  (0.54 – 1.05) | 0.74†  (0.53 – 1.04) | 0.73†  (0.52 – 1.02) | 0.77  (0.55 – 1.08) | 0.78†  (0.59 – 1.03) | 0.79  (0.59 – 1.05) |
|  | 3 | 1.05  (0.76 – 1.45) | 1.05  (0.76 – 1.45) | 1.06  (0.76 – 1.46) | 1.03  (0.74 – 1.43) | 1.07  (0.77 – 1.48) | 1.05  (0.80 – 1.37) | 1.07  (0.81 – 1.41) |
|  | 4 | 1.53**  (1.12 – 2.10) | 1.51*  (1.10 – 2.07) | 1.51*  (1.10 – 2.06) | 1.50*  (1.09 – 2.06) | 1.49*  (1.09 – 2.04) | 1.39*  (1.07 – 1.81) | 1.32*  (1.01 – 1.72) |
|  |  |  |  |  |  |  |  |  |
| Neighborhood deprivation (age 5) | 1 | Ref | Ref | Ref | Ref | Ref | Ref | Ref |
|  | 2 | 1.39  (0.74 – 2.60) | 1.37  (0.73 – 2.57) | 1.38  (0.74 – 2.58) | 1.41  (0.76 – 2.62) | 1.33  (0.74 – 2.39) | 1.26  (0.75 – 2.12) | 1.20  (0.74 – 1.97) |
|  | 3 | 1.26  (0.90 – 1.77) | 1.26  (0.90 – 1.77) | 1.29  (0.91 – 1.81) | 1.25  (0.89 – 1.75) | 1.27  (0.90 – 1.78) | 1.19  (0.89 – 1.59) | 1.21  (0.91 – 1.62) |
|  | 4 | 1.55*  (1.05 – 2.30) | 1.56*  (1.05 – 2.30) | 1.53*  (1.03 – 2.26) | 1.52*  (1.03 – 2.25) | 1.52*  (1.02 – 2.27) | 1.43*  (1.03 – 1.98) | 1.39†  (1.00 – 1.95) |
|  | 5 | 1.83***  (1.33 – 2.53) | 1.81***  (1.31 – 2.49) | 1.83***  (1.32 – 2.52) | 1.80***  (1.31 – 2.48) | 1.78***  (1.29 – 2.45) | 1.72***  (1.31 – 2.26) | 1.68***  (1.27 – 2.21) |
|  |  |  |  |  |  |  |  |  |
| Neighborhood deprivation (age 12) | 1 | Ref | Ref | Ref | Ref | Ref | Ref | Ref |
|  | 2 | 1.93*  (1.11 – 3.36) | 1.82*  (1.04 – 3.20) | 1.84*  (1.06 – 3.17) | 1.95*  (1.12 – 3.39) | 1.81*  (1.06 – 3.08) | 1.58*  (1.01 – 2.46) | 1.44  (0.92 – 2.24) |
|  | 3 | 1.51*  (1.09 – 2.10) | 1.51*  (1.09 – 2.09) | 1.51*  (1.09 – 2.10) | 1.51*  (1.09 – 2.10) | 1.48*  (1.06 – 2.05) | 1.44*  (1.09 – 1.89) | 1.44*  (1.09 – 1.90) |
|  | 4 | 1.60*  (1.07 – 2.39) | 1.56*  (1.04 – 2.33) | 1.57*  (1.05 – 2.35) | 1.54*  (1.03 – 2.29) | 1.52*  (1.01 – 2.28) | 1.50*  (1.07 – 2.11) | 1.40†  (0.99 – 1.99) |
|  | 5 | 2.18***  (1.58 – 3.00) | 2.14***  (1.55 – 2.95) | 2.13***  (1.55 – 2.95) | 2.11***  (1.53 – 2.91) | 2.07***  (1.50 – 2.84) | 2.02***  (1.54 – 2.65) | 1.92***  (1.46 – 1.52) |
|  |  |  |  |  |  |  |  |  |
| Neighborhood disorder | 1 | Ref | Ref | Ref | Ref | Ref | Ref | Ref |
|  | 2 | 1.38*  (1.03 – 1.85) | 1.37*  (1.03 – 1.83) | 1.39*  (1.04 – 1.85) | 1.37*  (1.03 – 1.84 | 1.40*  (1.05 – 1.87) | 1.35*  (1.06 – 1.71) | 1.38*  (1.08 – 1.76) |
|  | 3 | 1.66***  (1.25 – 2.21) | 1.63**  (1.23 – 2.17) | 1.63**  (1.23 – 2.17) | 1.62**  (1.22 – 2.15) | 1.59**  (1.20 – 2.11) | 1.56***  (1.24 – 1.98) | 1.48**  (1.17 – 1.88) |
|  |  |  |  |  |  |  |  |  |
| Neighborhood social cohesion | 1 | Ref | Ref | Ref | Ref | Ref | Ref | Ref |
|  | 2 | 1.07  (0.81 – 1.41) | 1.05  (0.79 – 1.39) | 1.06  (0.80 – 1.40) | 1.06  (0.80 – 1.41) | 1.06  (0.80 – 1.40) | 1.06  (0.84 – 1.34) | 1.04  (0.81 – 1.32) |
|  | 3 | 1.63**  (1.22 – 2.16) | 1.60**  (1.21 – 2.13) | 1.59**  (1.19 – 2.11) | 1.62**  (1.22 – 2.15) | 1.59**  (1.19 – 2.11) | 1.54***  (1.22 – 1.95) | 1.49**  (1.17 – 1.89) |
|  |  |  |  |  |  |  |  |  |
| Residential mobility | 1 | Ref | Ref | Ref | Ref | Ref | Ref | Ref |
|  | 2 | 1.06  (0.80 – 1.40) | 1.05  (0.79 – 1.39) | 1.07  (0.81 – 1.42) | 1.04  (0.79 – 1.38_ | 0.99  (0.75 – 1.32) | 1.05  (0.83 – 1.33) | 1.00  (0.79 – 1.28) |
|  | 3 | 1.33*  (1.00 – 1.77) | 1.30†  (0.98 – 1.73) | 1.30†  (0.97 – 1.73) | 1.30†  (0.98 – 1.73) | 1.18  (0.88 – 1.58) | 1.23†  (0.98 – 1.56) | 1.09  (0.85 – 1.39) |
|  |  |  |  |  |  |  |  |  |
| Family poverty | 1 | Ref | Ref | Ref | Ref | Ref | Ref | Ref |
|  | 2 | 1.63**  (1.21 – 2.18) | 1.62**  (1.21 – 2.17) | 1.61**  (1.20 – 2.16) | 1.60**  (1.20 – 2.15) | 1.62**  (1.21 – 2.17) | 1.56***  (1.22 – 1.99) | 1.54**  (1.20 – 1.98) |
|  | 3 | 2.20***  (1.66 – 2.91) | 2.17***  (1.64 – 2.87) | 2.17***  (1.64 – 2.87) | 2.11***  (1.60 – 2.80) | 2.04***  (1.54 – 2.71) | 2.04***  (1.61 – 2.58) | 1.88***  (1.48 – 2.39) |

Note: CI, confidence intervals; OR, odds ratio from ordinal logistic regression; Ref, reference category; † p<0.1; * p<0.05; ** p<0.01; *** p<0.001. ^a^ Levels of environmental risks were: Urbanicity, 1-3=rural to urban; Air pollution, 1-4=lowest to highest quartile; Neighborhood deprivation, 1-5=lowest to highest quintile; Neighborhood disorder, 1-3=lowest to highest tertile; Neighborhood social cohesion, 1-3=highest to lowest tertile; Residential mobility, 1-3=0, 1, 2+ residential moves; Family poverty, 1-3=high to low socioeconomic status. Aside from the unadjusted model, all models control for sex in addition to the specified genetic risk measure. All analyses control for the non-independence of twin observations and were conducted on complete cases where participants had full data in the fully adjusted model: N=1,610–1,789.

**Supplementary Table 4:** Performance statistics of CMAQ-Urban for 2012.

| **Pollutant** | **Number of data** | **Observed mean (µg/m^3^)** | **Modelled mean (µg/m^3^)** | **FAC2** | **MB (µg/m^3^)** | **NMB** | **RMSE (µg/m^3^)** | **r** |
| --- | --- | --- | --- | --- | --- | --- | --- | --- |
| NO_2_ | 109 | 37.75 | 38.90 | 0.96 | 1.14 | 0.03 | 10.32 | 0.90 |

Note: FAC2, fraction of predictions within a factor of two; MB, mean bias; NMB, normalised mean bias; NO_2_, nitrogen dioxide; RMSE, root mean squared error; r, correlation coefficient.

**References from Supplementary Materials**

Abel G. A., Barclay M. E. and Payne R. A. (2016) Adjusted indices of multiple deprivation to enable comparisons within and between constituent countries of the UK including an illustration using mortality rates. *BMJ Open, 6*, e012750. doi:10.1136/bmjopen-2016-012750

Beevers S. D., Kitwiroon N., Williams M. L. and Carslaw D. C. (2012) One way coupling of CMAQ and a road source dispersion model for fine scale air pollution predictions. *Atmospheric Environment, 59*, 47-58. doi:10.1016/j.atmosenv.2012.05.034

Bennett N., Jarvis L., Rowlands O., Singleton N. and Haselden L. (1996). *Living in Britain: Results from the 1994 general household survey*. London: HMSO.

CACI Information Services. (2006). *ACORN User Guide*. London, UK: CACI.

Carslaw D. C. (2011, April 15). Defra urban model evaluation analysis - Phase 1. *Report to Defra, Environmental Research Group*. Retrieved from <https://uk-air.defra.gov.uk/library/reports?report_id=654>.

Caspi A., Taylor A., Moffitt T. E. and Plomin R. (2000) Neighborhood deprivation affects children's mental health: Environmental risks identified in a genetic design. *Psychological Science, 11*, 338-342. doi:doi.org/10.1111/1467-9280.00267

Department for Communities and Local Government. (2015). *The English Indices of Deprivation: 2015*. London: Department for Communities and Local Government.

Maynard R. A. (1997). *Kids having kids: Economic costs & social consequences of teen pregnancy*. Washington, DC: The Urban Insitute.

Messner S. F., Baumer E. P. and Rosenfeld R. (2004) Dimensions of social capital and rates of criminal homicide. *American Sociological Review, 69*, 882-903. doi:10.1177/000312240406900607

Miller T. J., Cicchetti D., Markovich P. J., McGlashan T. H. and Woods S. W. (2004) The SIPS-Screen: a brief self-report screen to detect the schizophrenia prodrome. *Schizophrenia Research, 70 (suppl1)*, 78.

Miller T. J., McGlashan T. H., Rosen J. L., Cadenhead K., Ventura J., McFarlane W., . . . Woods S. W. (2003) Prodromal assessment with the structured interview for prodromal syndromes and the scale of prodromal symptoms: predictive validity, interrater reliability, and training to reliability. *Schizophrenia Bulletin, 29*, 703.

Moffitt T. E. and The E-Risk Study Team (2002) Teen-aged mothers in contemporary Britain. *Journal of Child Psychology and Psychiatry and Allied Disciplines, 43*, 727-742. doi:10.1111/1469-7610.00082

Odgers C. L., Caspi A., Bates C. J., Sampson R. J. and Moffitt T. E. (2012a) Systematic social observation of children's neighborhoods using Google Street View: A reliable and cost-effective method. *Journal of Child Psychology and Psychiatry and Allied Disciplines, 53*, 1009-1017. doi:10.1111/j.1469-7610.2012.02565.x

Odgers C. L., Caspi A., Russell M. A., Sampson R. J., Arseneault L. and Moffitt T. E. (2012b) Supportive parenting mediates neighborhood socioeconomic disparities in children's antisocial behavior from ages 5 to 12. *Development and Psychopathology, 24*, 705-721. doi:doi:10.1017/S0954579412000326

Odgers C. L., Moffitt T. E., Tach L. M., Sampson R. J., Taylor A., Matthews C. L. and Caspi A. (2009) The protective effects of neighborhood collective efficacy on British children growing up in deprivation: A developmental analysis. *Developmental Psychology, 45*, 942-957. doi:10.1037/a0016162

Office for National Statistics. (2013, August 28). Urban and rural area definitions for policy purposes in England and Wales: Methodology (v1.0). Retrieved from <https://assets.publishing.service.gov.uk/government/uploads/system/uploads/attachment_data/file/239477/RUC11methodologypaperaug_28_Aug.pdf>.

Poulton R., Caspi A., Moffitt T. E., Cannon M., Murray R. and Harrington H. (2000) Children's self-reported psychotic symptoms and adult schizophreniform disorder: A 15-year longitudinal study. *Archives of General Psychiatry, 57*, 1053-1058. doi:10.1001/archpsyc.57.11.1053

Price T. S., Freeman B., Craig I., Petrill S. A., Ebersole L. and Plomin R. (2000) Infant zygosity can be assigned by parental report questionnaire data. *Twin Research and Human Genetics, 3*, 129-133. doi:10.1375/twin.3.3.129

Sampson R. J. and Raudenbush S. W. (1999) Systematic social observation of public spaces: A new look at disorder in urban neighborhoods. *American Journal of Sociology, 105*, 603-651. doi:10.1086/210356

Sampson R. J., Raudenbush S. W. and Earls F. (1997) Neighborhoods and violent crime: A multilevel study of collective efficacy. *Science, 277*, 918-924. doi:10.1126/science.277.5328.918

Schreier A., Wolke D., Thomas K., Horwood J., Hollis C., Gunnell D., . . . Duffy L. (2009) Prospective study of peer victimization in childhood and psychotic symptoms in a nonclinical population at age 12 years. *Archives of General Psychiatry, 66*, 527-536. doi:10.1001/archgenpsychiatry.2009.23

Spauwen J., Krabbendam L., Lieb R., Wittchen H. U. and van Os J. (2004) Does urbanicity shift the population expression of psychosis? *Journal of Psychiatric Research, 38*, 613-618. doi:10.1016/j.jpsychires.2004.04.003

Trouton A., Spinath F. M. and Plomin R. (2002) Twins early development study (TEDS): A multivariate, longitudinal genetic investigation of language, cognition and behavior problems in childhood. *Twin Research, 5*, 444-448. doi:10.1375/twin.5.5.444

Trzesniewski K. H., Moffitt T. E., Caspi A., Taylor A. and Maughan B. (2006) Revisiting the association between reading achievement and antisocial behavior: New evidence of an environmental explanation from a twin study. *Child Development, 77*, 72-88. doi:10.1111/j.1467-8624.2006.00857.x

Yoshizumi T., Murase S., Honjo S., Kaneko H. and Murakami T. (2004) Hallucinatory experiences in a community sample of Japanese children. *Journal of the American Academy of Child and Adolescent Psychiatry, 43*, 1030-1036. doi:10.1097/01.chi.0000126937.44875.6b

Yung A. R., Nelson B., Baker K., Buckby J. A., Baksheev G. and Cosgrave E. M. (2009) Psychotic-like experiences in a community sample of adolescents: Implications for the continuum model of psychosis and prediction of schizophrenia. *Australian and New Zealand Journal of Psychiatry, 43*, 118-128. doi:10.1080/00048670802607188
